# The νSaα Specific Lipoprotein Like Cluster (*lpl*) of *S*. *aureus* USA300 Contributes to Immune Stimulation and Invasion in Human Cells

**DOI:** 10.1371/journal.ppat.1004984

**Published:** 2015-06-17

**Authors:** Minh Thu Nguyen, Beatrice Kraft, Wenqi Yu, Dogan Doruk Demicrioglu, Tobias Hertlein, Marc Burian, Mathias Schmaler, Klaus Boller, Isabelle Bekeredjian-Ding, Knut Ohlsen, Birgit Schittek, Friedrich Götz

**Affiliations:** 1 Department of Microbial Genetics, University of Tübingen, Tübingen, Germany; 2 Department of Dermatology, University of Tübingen, Tübingen, Germany; 3 Institute for Molecular Infection Biology, University of Würzburg, Würzburg, Germany; 4 Immunoregulation, Department of Biomedicine, University of Basel and University Hospital Basel, Basel, Switzerland; 5 Paul-Ehrlich-Institut, Federal regulatory agency for Vaccines and Biomedicines, Langen, Germany; 6 Institute of Medical Microbiology, Immunology, and Parasitology, University Hospital Bonn, Bonn, Germany; National Institutes of Health, UNITED STATES

## Abstract

All *Staphylococcus aureus* genomes contain a genomic island, which is termed νSaα and characterized by two clusters of tandem repeat sequences, i.e. the exotoxin (*set*) and 'lipoprotein-like' genes (*lpl*). Based on their structural similarities the νSaα islands have been classified as type I to IV. The genomes of highly pathogenic and particularly epidemic *S*. *aureus* strains (USA300, N315, Mu50, NCTC8325, Newman, COL, JH1 or JH9) belonging to the clonal complexes *CC5* and *CC8* bear a type I νSaα island. Since the contribution of the *lpl* gene cluster encoded in the νSaα island to virulence is unclear to date, we deleted the entire *lpl* gene cluster in *S*. *aureus* USA300. The results showed that the mutant was deficient in the stimulation of pro-inflammatory cytokines in human monocytes, macrophages and keratinocytes. Purified lipoprotein Lpl1 was further shown to elicit a TLR2-dependent response. Furthermore, heterologous expression of the USA300 *lpl* cluster in other *S*. *aureus* strains enhanced their immune stimulatory activity. Most importantly, the *lpl* cluster contributed to invasion of *S*. *aureus* into human keratinocytes and mouse skin and the non-invasive *S*. *carnosus* expressing the *lpl* gene cluster became invasive. Additionally, in a murine kidney abscess model the bacterial burden in the kidneys was higher in wild type than in mutant mice. In this infection model the *lpl* cluster, thus, contributes to virulence. The present report is one of the first studies addressing the role of the νSaα encoded *lpl* gene cluster in staphylococcal virulence. The finding that the *lpl* gene cluster contributes to internalization into non-professional antigen presenting cells such as keratinocytes highlights the *lpl* as a new cell surface component that triggers host cell invasion by *S*. *aureus*. Increased invasion in murine skin and an increased bacterial burden in a murine kidney abscess model suggest that the *lpl* gene cluster serves as an important virulence factor.

## Author Summary

Highly pathogenic and epidemic *Staphylococcus aureus* strains carry a pathogenicity island in their genome that contains a cluster of lipoprotein-encoding genes termed *lpl*. As the role *lpl* in virulence is still unclear, we deleted the entire *lpl* cluster in the community-acquired methicillin-resistant *S*. *aureus* (CA-MRSA) USA300 and found that the mutant was defective in stimulation of pro-inflammatory cytokines in human immune cells. Moreover, the major finding highlighted in this study is that the *lpl* cluster contributes to invasion into non-professional phagocytes such as epithelial cells and keratinocytes. Furthermore, the *lpl*-dependent increase in invasive activity, most likely, accounts for the enhanced bacterial burden observed in a murine kidney abscess model. In general, internalization of a pathogen into host epithelial cells shields the pathogen from immune defense and antibiotic treatment. However, further investigation is needed to clarify whether the increased ability to invade host cells is responsible for the potent disseminative activity and hypervirulent phenotype characterizing the νSaα type I island expressing *S*. *aureus* strains, including the USA300 CA-MRSA strain.

## Introduction

In *Staphylococcus aureus*, the lipid moiety of lipoproteins represents the major signal activating innate immunity. This was confirmed by the fact that innate immune activation was significantly decreased in diacylglyceryl transferase enzyme encoding gene (*lgt*) deleted mutants [1]. The previously described stimulatory activity of lipoteichoic acid (LTA) was, thus, most likely due to contamination of the LTA fraction with highly active natural lipoproteins and/or lipopeptides [2,3].

One of the first lipoproteins to be analyzed and described in *S*. *aureus* was the membrane bound penicillinase [4]. The search of the *S*. *aureus* genomes for the lipobox motif revealed approximately 55–70 genes encoding putative lipoproteins [1,5]. Many of them exert crucial functions as transporters (iron, manganese, nickel, zinc, amino acid, oligopeptide, glycine betaine, sugar, teichoic acid transporter, or preprotein translocase subunit YidC) or chaperons, e.g. phage terminases, hem/copper-type cytochrome/quinol oxidase, pyruvate-formate-lyase-activating enzyme, protein-disulfide isomerase, or peptidyl-prolyl *cis/trans* isomerase (PrsA) [1]. It was, therefore, not surprising that the *lgt* mutation not only affected high-affinity metal ion uptake but mutants were also attenuated in virulence [6].

**Fig 1:**
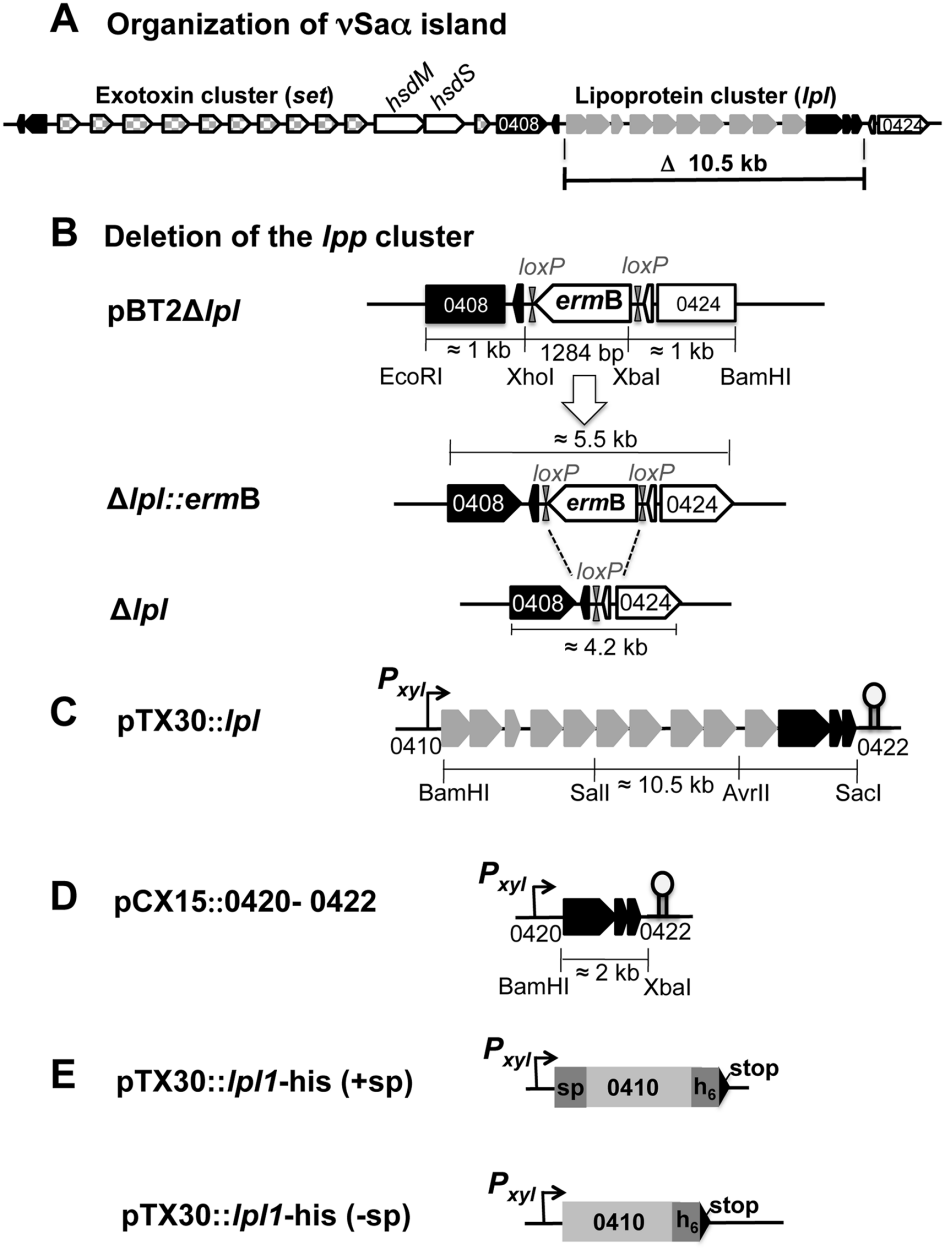
Schematic illustration of USA300 specific νSaα island and construction of *lpl* operon deletion mutants and of complementing plasmids. (A) Genetic organization of the exotoxin (*set*) and lipoprotein (*lpl*) cluster of νSaα. Both clusters are separated by a DNA methylase unit composed of *hsdM* (methylase) and *hsdS* (target selection). The function of the black-marked genes is unknown. The sequence of the two rho-independent transcription terminators, Ω1 and Ω2 are: Ω1(ACTACTAAATAAAGCGACCAATGTTCAGTATATTCACAACTGACACTAGGCCGCTTc**TTTTT**AATTTATA: kcal -5.45) and for Ω2 (TTTATCCATAGGGCTAGGACATGTATGTGTCTTAGTCC **TTTTT**ATATTTA: kcal -8.30), the underlined sequences represent the stem region. For terminator analysis the ARNold program combining two complementary programs, Erpin and RNA motif (Lesnik, 2001 #53, Gautheret, 2001 #52) was used. (B) Illustrates the steps in creating a *lpl* deletion in USA300. pBT2Δ*lpl* represents the knock-out plasmid where the *lpl* genes 0408–0419 plus the adjacent genes 0420–0422 were substituted by *erm*B (erythromycin resistance cassette) flanked by *loxP* sequences for later deletion of *erm*B by Cre recombinase. For homologous double-cross recombination *ermB* is additionally flanked by roughly 1 kb upstream and downstream DNA-sequences. The chromosomal site of the *lpl*-deletion mutants with and without *ermB* (after Cre-*loxP* recombination) is shown for Δ*lpl*::*erm*B and Δ*lpl*, respectively. (C) pTX30::*lpl* is the *lpl* complementation plasmid in which the transcription of the genes 0410 to 0422 is xylose-inducible. (D) pCX15::0420–0422 is the complementation plasmid for the three *lpl* downstream genes of unknown function; they are also xylose inducible. (E) pTX30*lpl1*-his (+sp/-sp, denotes the presence and absence of the lipo signal sequence) is the xylose-inducible expression plasmid for the first lipoprotein gene, *lpl1* (0410), the *lpl1* sequence is extended 3' by 6 histidine and a stop codon.

Highly pathogenic and, in particular, epidemic *S*. *aureus* strains bear various genomic islands in their genome that encode prophages, toxins and antibiotic resistance genes, e.g. SCCmec, or tandem paralogous genes such as those encoded in the νSaα island. The term νSaα refers to non-phage and non-SCC genomic islands that are exclusively present in *S*. *aureus* and inserted at specific loci in the chromosome [7]. The genomic island termed νSaα is present in all *S*. *aureus* genomes sequenced to date [8–10] but is not found in coagulase-negative species, including the non-pathogenic species *S*. *carnosus* [11]. The genetic organization of νSaα is highly conserved and is composed of two gene clusters: one cluster encodes a number of highly homologous exotoxin-encoding genes (*set*), the other one encodes lipoproteins, referred to as 'lipoprotein-like' (*lpl*), with a typical lipo-box containing signal sequence [12]. The genetic organization of νSaα of *S*. *aureus* USA300 is illustrated in Fig 1A. The *set* and *lpl* clusters are separated by the *hsdM* and *hsdS* genes that encode a restriction-modification system. While HsdS selects the genomic target sequence, HsdM is serves as a methylase. It has been proposed that this system contributes to stabilization and maintenance of the *S*. *aureus* genomic islands. It was further suggested that the νSaα genomic islands originate from mobile genetic elements that were acquired independently through intra-species genetic transfer between *S*. *aureus* strains [8]. The finding that some νSaα types contain a remnant transposase gene supports this hypothesis. As the *hsdS* alleles correlate with the structural similarity of the islands its sequence has been used for classification of νSaα islands into type I to IV.

All *lpl* genes are sequentially lined up in the same orientation, thus comprising a paralog cluster. The *lpl* genes within one type are highly homologous (almost 99%), but are significantly different from those encoded in other types. The various *lpl* genes within one cluster are further distinguished by a 5′- and 3′-variable region and a highly conserved intragenic region, which might represent a structural prerequisite for gene shuffling, tandem duplications and diversification [7]. Homologs of these *lpl* genes were also found in loci other than the genomic island and represent the largest group of paralogous genes in various *S*. *aureus* strains [8,10].

The *S*. *aureus* strains USA300, N315, Mu50, NCTC8325, Newman, COL, JH1 and JH9 possess a type I νSaα island [8]. Interestingly, all of these strains belong to the clonal complex 5 and 8 (*CC5 & CC8*) whose representatives are characterized by high transmissibility and virulence. Although the genome of USA300 is known [9], the molecular genetic basis of the high transmissibility and hypervirulence of the CC8 strains and USA300, in particular, is unknown. However, the broad distribution and apparent persistence of the νSaα island in *S*. *aureus* suggests a potential function in virulence and, additionally, in the dissemination of epidemic strains. We, therefore, carried out our studies in *S*. *aureus* USA300, a community-associated MRSA (CA-MRSA) that is epidemic in the general population and has been associated with necrotizing fasciitis, pneumonia and other rapidly progressing and life-threatening infections [9,13]. According to multilocus sequence typing (MLST) USA300 belongs to the clonal complex CC8, which contains many strains with high clinical impact [14,15].

Since the role the *lpl* gene cluster of the νSaα islands in virulence is unclear to date, we investigated its function in *S*. *aureus* USA300 by deleting the entire *lpl* gene cluster. We found that the mutant was deficient in innate immune stimulation, host cell invasion and virulence.

## Results

### Genomic characterization of the νSaα specific lipoprotein like cluster (*lpl*) of *S*. *aureus* USA300

**Fig 2:**
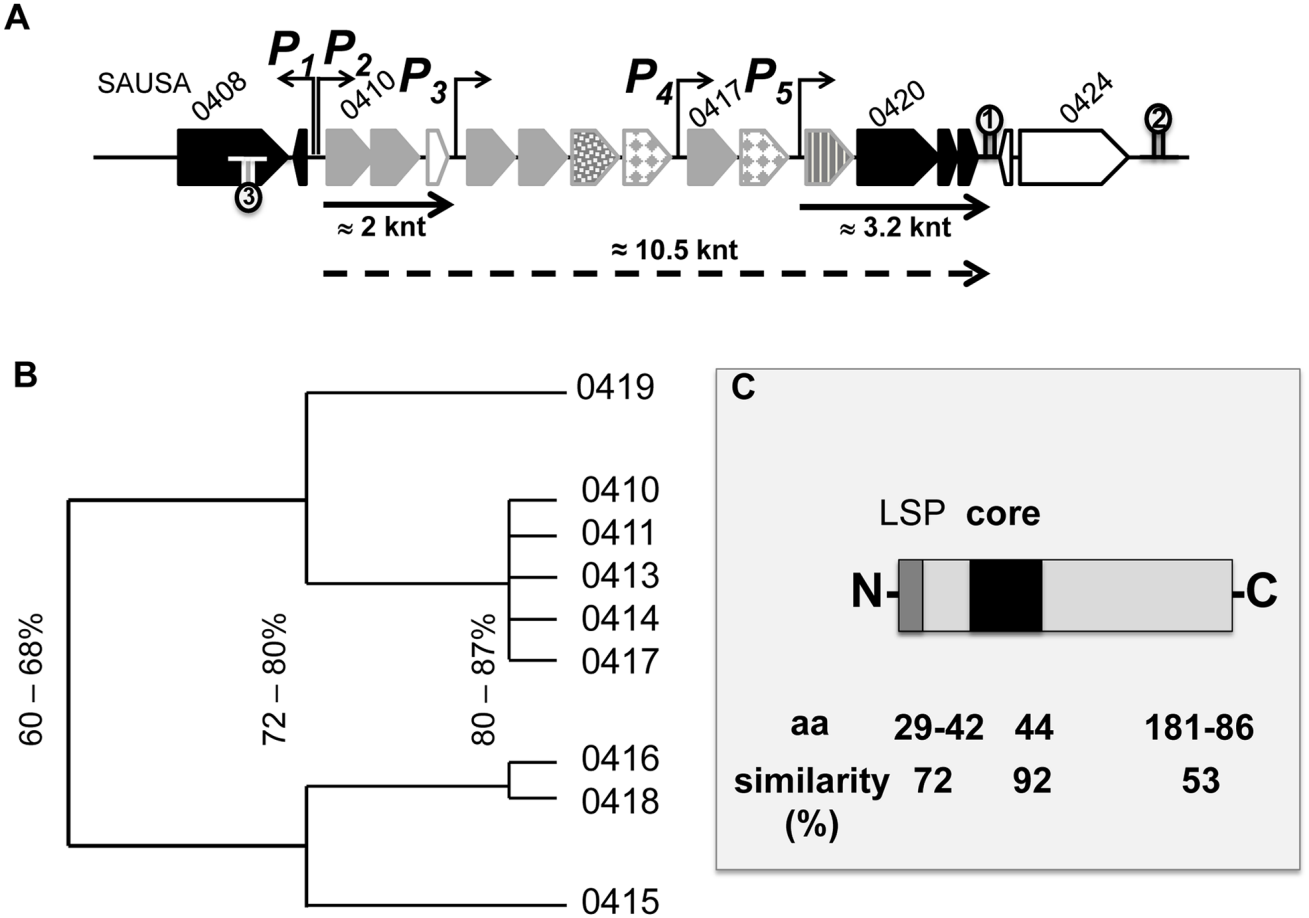
Genetic organization of the *lpl* cluster operon. (A) Shows a genomic section of the *lpl* cluster with five predicted promoters (P1-5) and three transcription termination sites (indicated by hair-pin symbols). The genes 0410–0422 comprise most likely an operon, as they are not interrupted by a transcription terminator sequence. The *lpl* operon is composed of ten genes (0410–0419) encoding lipoproteins (grayish arrows) and of three non-lipoprotein encoding genes 0420–0422 (black arrows). By Northernblot analysis two transcripts of 2 and 3.2 knt could be verified; most likely there is a 10.5 knt read-through transcript for the entire *lpl* operon (indicated by dotted arrow). (B) Shows a cluster dendrogram based on the sequence similarity of lipoproteins (Lpl). (C) Alignment of all Lpl proteins revealed a highly conserved (92%) core sequence while the flanking sequences are less conserved. LSP, lipo signal peptide.

The lipoprotein-like cluster within the νSaα island of USA300 comprises ten *lpl* genes (*lpl* 0410–0419). All of them contain a classical lipo-box motif in the signal sequence (Fig 2A). Based on protein sequence similarity the corresponding Lpl proteins can be grouped into three clusters: Lpls encoded by the genes 0410, 0411, 0413, 0414, 0417 share > 80% similarity; the two Lpls encoded by 0416 and 0418 also share > 80% similarity but the similarity between 0410 and 0416 is only approximately 60% (Fig 2B). The similarity of the Lpls suggests that they might have redundant functions. The *lpl* cluster contains several potential promoters located upstream of the genes 0410, 0413, 0417 and 0419 (Fig 2A). By Northern blot analysis (S1 Fig) we could verify the transcripts starting from P2, P4 and P5 promoters. However, we assume that a run-through transcript of approximately 10.5 knt is generated because there is no conspicuous transcription terminator sequence in the *lpl* cluster (Fig 2A). There are two predicted rho-independent transcription terminators, Ω1 and Ω2, downstream of 0422 and 0424, respectively. Downstream of the *lpl* cluster, but upstream of the Ω2, there are three genes (0420–0422) with unknown function. These highly conserved genes are present in all presently sequenced νSaα islands. We, therefore, predict that they might be involved in Lpl biosynthesis/modification. The third transcription terminator, Ω3, is inserted in opposite direction, downstream of 0409 (Fig 2A).

To study the potential role of the νSaα-specific *lpl* cluster in USA300 we constructed a marker-less deletion mutant, USA300Δ*lpl*, in which the entire *lpl* operon was deleted including the three downstream genes (0420–0422) of unknown function (Fig 1B). For complementation of the mutant two plasmids were constructed: pTX30::*lpl* contains all the genes (0410–0422) that had been deleted (Fig 1C), and pCX15::0420–0422 contains only the three non-lipoprotein genes (Fig 1D); in these two compatible plasmids the genes are expressed under the control of a xylose-inducible promoter.

### The USA300 νSaα-specific *lpl* cluster enhances TNF-α and IL-6 production in monocytes

**Fig 3:**
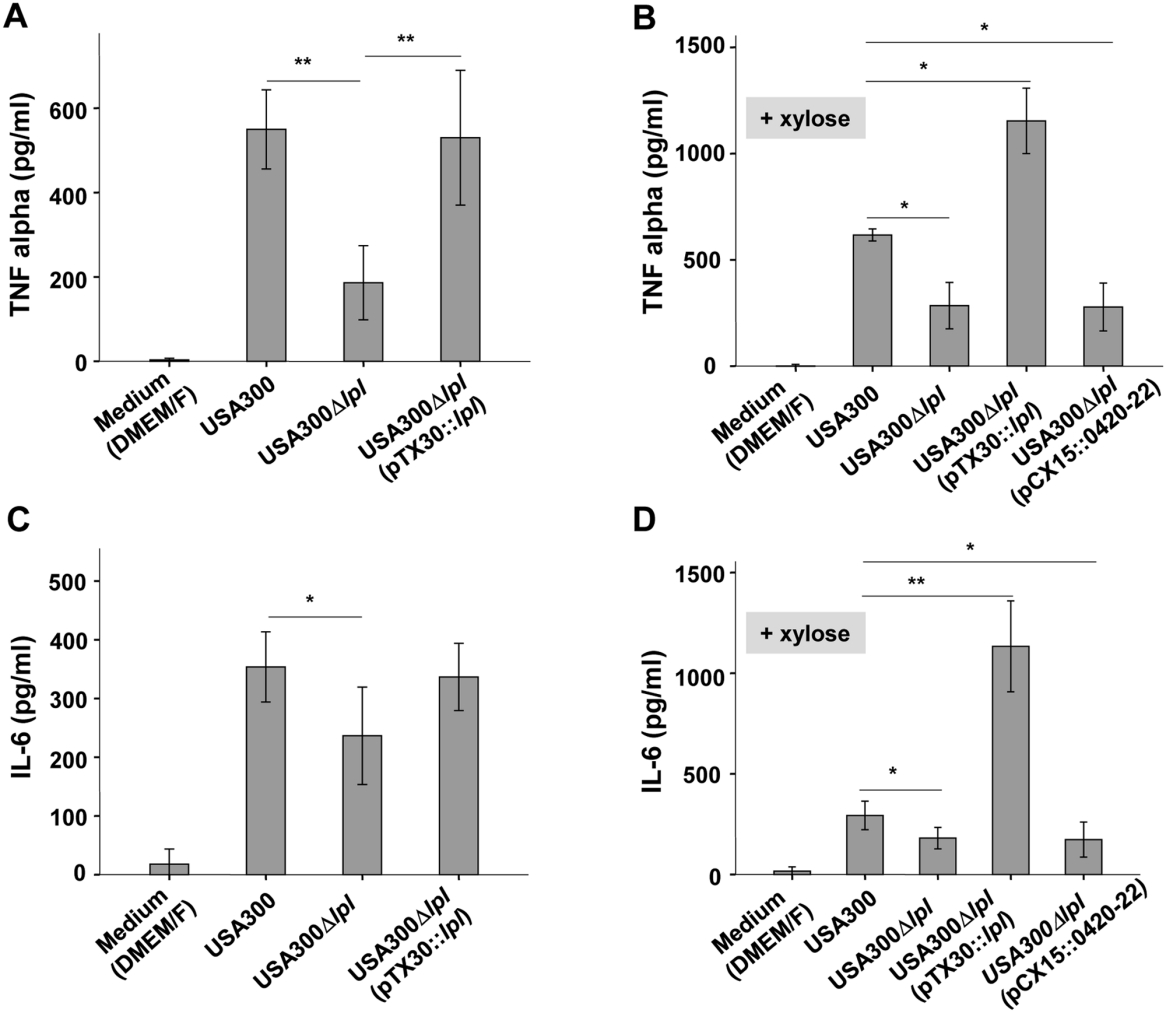
Induction of TNF-α and IL-6 by Mono Mac 6 cells upon infected with USA300, its Δ*lpl* deletion mutant and complemented mutant. USA300, Δ*lpl*, Δ*lpl* (pTX30::*lpl*) and Δ*lpl* (pCX15::0420–0422) were cultured in TSB medium (A and C) and TSB+0.8% xylose (B and D) for 16 hours. For immune stimulation 10^6^ Mono Mac 6 cells were infected with a MOI of 30:1. Released TNF-α and IL-6 into the supernatant was determined by ELISA after 4 h and 24 h of stimulation. The experiments in duplicate were conducted at least 3 times. Error bars indicate standard error. Statistical significances were calculated by using Student's t-tests or analysis of variance (ANOVA): not significant P>0.05, * P<0.05, ** P <0.01.

In USA300 the νSaα specific *lpl* genes represent roughly 16% of all annotated lipoproteins. To investigate whether the *lpl* cluster contributes to the stimulation of innate immune cells, the human monocytic cell line (Mono Mac 6) was infected with an MOI of 30:1 with living cells of the parental strain USA300 wt, the *lpl* deletion mutant Δ*lpl*, and the complemented mutant Δ*lpl* (pTX30::*lpl*). The production of pro-inflammatory cytokines such as TNF-α and IL-6 was determined after 4 and 24 h of stimulation. The stimulatory conditions for TNF-α and IL-6 were selected based on prior experiments (S2 and S3 Figs). The results showed that the production of TNF-α and IL-6 was significantly decreased when stimulating with the Δ*lpl* mutant compared to the wt: TNF-α decreased by roughly 70% (Fig 3A) and IL-6 by approximately 40% (Fig 3C). In the complemented mutant the cytokine levels were restored to the parental levels, even in the absence of xylose.

However, when the complemented mutant USA300*lpl* (pTX30::*lpl*) was cultivated in the presence of 0.8% xylose TNF-α and IL-6 production was increased 2- to 3-fold, respectively, when compared to the parental strain (Fig 3B and 3D). In order to investigate whether the three non-lipoprotein genes (0420–0422) downstream of the *lpl* cluster, which are part of the *lpl* operon, had an immune modulatory effect, USA300*lpl* (pCX15::0420–22) was constructed. However, the xylose-induced genes 0420–0422 showed no effect on TNF-α and IL-6 production (Fig 3B and 3D), suggesting that only the Lpl proteins contribute to TNF-α and IL-6 production. Similar results were obtained with different MOIs tested (S2 Fig).

**Fig 4:**
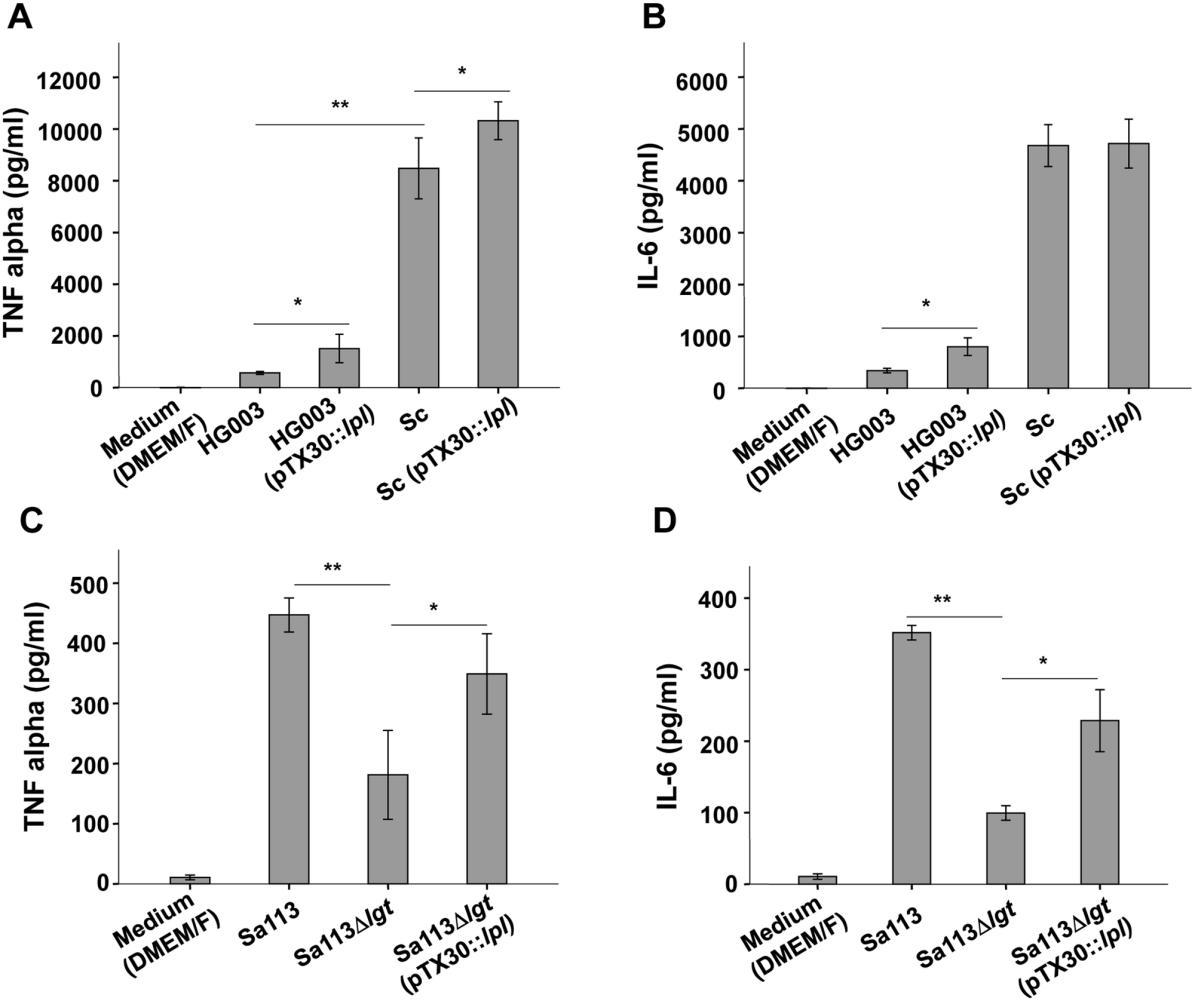
Induction of TNF-α, IL-6 by Mono Mac 6 cells infected with various staphylococcal strains. Staphylococcal strains were cultured in TSB+0.8% xylose for 16 h and used to infect 10^6^ Mono Mac 6 cells with a MOI of 30:1. TNF-α and IL-6 were determined after 4 h and 24 h of stimulation. Production of TNF-α (A) and IL-6 (B) by Mono Mac 6 cells infected with *S*. *aureus* HG003, HG003 (pTX30::*lpl*), *S*. *carnosus* TM300 (Sc), and *Sc* (*pTX30*::*lpl*). Production of TNF-α (C) and IL-6 (D) by Mono Mac 6 cells infected with *S*. *aureus* SA113, SA113Δ*lgt* and SA113Δ*lgt* (pTX30::*lpl*). The experiments in duplicate were conducted at least 3 times. Error bars indicate standard error. Statistical significances were calculated by using Student's t-tests or analysis of variance (ANOVA): not significant P>0.05, * P<0.05, ** P <0.01.

We next investigated whether the *lpl*-cluster exerts a similar immune stimulatory effect in other staphylococcal strains. To this end we cloned pTX30::*lpl* into *S*. *aureus* HG003, which is a *rsbU*- and *tcaR*-repaired derivate of NCTC8325 [16] and into *S*. *carnosus* TM300, a non-pathogenic foodborne staphylococcal species [11,17]. The results showed that in HG003, as in USA300, xylose-induced expression of the *lpl*-cluster led to an approximately four-fold induction of TNF-α and IL-6 production (Fig 4A and 4B). In *S*. *carnosus* (pTX30::*lpl*) TNF-α production was only slightly increased and for IL-6 production no difference was observed (Fig 4A and 4B). However, one of the most remarkable differences between the pathogenic *S*. *aureus* strains and the non-pathogenic *S*. *carnosus* strain was the generally much higher activation of Mono Mac 6 by *S*. *carnosus*. While USA300 and HG003 triggered TNF-α production in a range of 500 pg/ml, *S*. *carnosus* TM300-derived TNF-α production was increased to > 8000 pg/ml, which is 16 times higher than that of the *S*. *aureus* strains. A similar difference was observed with IL-6 (Fig 4A and 4B). Thus, *S*. *carnosus* has a much higher TLR2-dependent immune stimulatory activity in Mono Mac 6 than USA300.

We hypothesize that the ability of *S*. *carnosus* to mount a strong TNF-α and IL-6 response is due to its non-pathogenicity. As a consequence it can rapidly be neutralized by a robust immune response. Alternatively, *S*. *carnosus* could be less toxic to Mono Mac 6 cells compared to *S*. *aureus* resulting in higher viable cell numbers that produce TNF-α/IL-6. We, therefore, checked the number of live Mono Mac 6 cells after stimulation with *S*. *aureus* and *S*. *carnosus* but observed no significant difference in cell viabilities (S4 Fig).

### The *lpl* cluster enhances cytokine and antimicrobial peptide expression in differentiated primary human keratinocytes and macrophages

**Fig 5:**
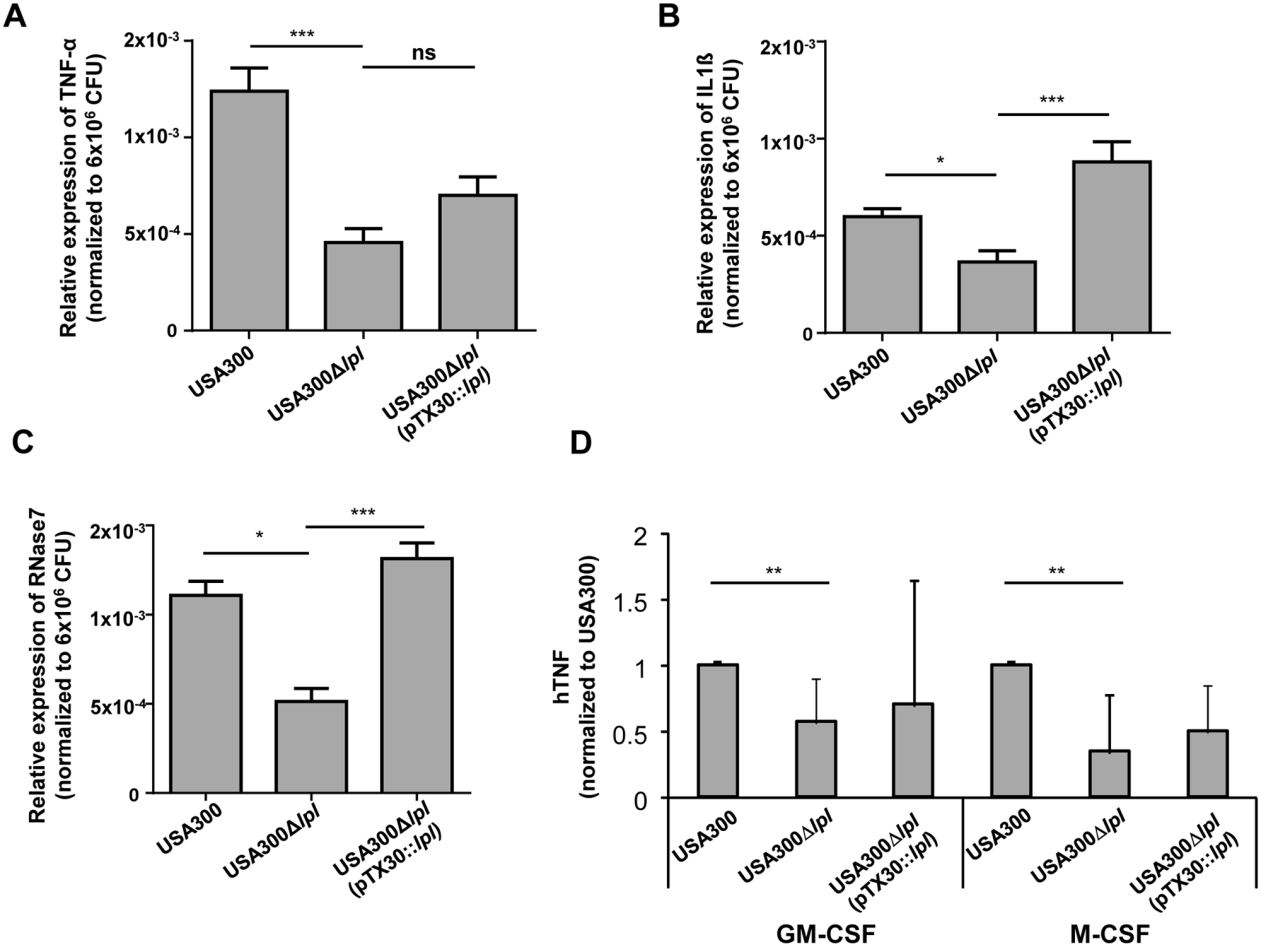
Cytokine and antimicrobial peptide (AMP) expression in differentiated primary human keratinocytes and macrophages. (A-C) Differentiated primary human keratinocytes were infected with USA300, its Δ*lpl*, and complemented mutant Δ*lpl* (pTX30::*lpl*) with a MOI of 30 (± 10) for 8 h. RNA expression of the cytokines TNF-α and IL-1β and the antimicrobial peptide RNase7 was analyzed by RT-PCR. Relative expression of target gene to reference gene (β-actin) is shown, normalized to 6x10^6^ CFU to compensate variations of MOI. Statistical analysis was performed using analysis of variance (ANOVA), n = 8; error bars indicate standard error of the mean (SEM). (D) Macrophages were generated from peripheral blood monocytes in the presence of GM-CSF (left) or M-CSF (right) for 5 days. Macrophages were stimulated with USA300 or its *lpl* mutant (MOI = 10) and supernatants harvested after 24 hours. TNF-α secretion was analyzed by ELISA. To limit donor variability results were normalized to USA300 = 100% (100% corresponds to 120 ± 113 (GM-CSF) and 73 ± 102 pg/ml (M-CSF)). The graph displays the average values ±SD obtained from n = 8 (GM-CSF) and n = 7 experiments (M-CSF). Statistical significances: not significant P>0.05; * P<0.05; ** P<0.01, ***P<0.001.

Differentiated primary human keratinocytes were infected with USA300, the *Δlpl* mutant, and the complemented mutant *Δlpl* (pTX30::*lpl*) with an MOI of 30 (± 10) for 8 h. mRNA expression of cytokines (TNF-α and IFN-γ) and the antimicrobial protein RNase7 was analyzed by RT-PCR. The relative expression of the target genes (*TNF-α*, *IL-1β* and *RNase7*) was normalized to β-actin as house keeping gene. In all cases the *Δlpl* mutant induced significantly lower levels of TNF-α, IL-1β and RNase7 transcripts than the parental strain or the complemented mutant, suggesting that the *lpl* cluster contributes to the expression of inflammatory cytokines and antimicrobial peptides in primary human keratinocytes (Fig 5A, 5B and 5C). To evaluate these findings in primary human leukocytes, human macrophages were generated in the presence of GM-CSF or M-CSF, respectively. Similarly, we observed significantly lower TNF-α production with the *Δlpl* mutant in both human macrophage subsets (Fig 5D).

### Cytokine production induced by the *lpl* cluster is conserved in an *lgt* mutant

It is well known that the lipid-modification of lipoproteins is crucial for the activation of innate immune cells via TLR2 signaling. The *S*. *aureus* Δ*lgt* mutants lacks lipidation of pre-prolipoproteins because the prolipoprotein diacylglyceryl transferase is absent. *S*. *aureus* Δ*lgt* mutants were formerly not only completely deficient in lipidation of pre-prolipoproteins, but also induced significantly less proinflammatory cytokines and chemokines and were compromised in respect to virulence [1,2,6]. Due to the importance of the lipid-moiety of lipoproteins for immune stimulation we anticipated that expression of the *lpl*-cluster in SA113Δ*lgt* mutant would have no effect on proinflammatory cytokine expression because the corresponding Lpls lack the lipid-moiety that is crucial for the initiation of TLR2/MyD88-dependent signaling. However, expression of the *lpl*-cluster in SA113Δ*lgt* (pTX30::*lpl*) caused a nearly two-fold increase in TNF-α and IL-6 production, suggesting that, unexpectedly, not only the lipid-moiety but also the Lpl protein portion might possess stimulatory activity (Fig 4C and 4D). Subsequent experiments, however, revealed that the stimulatory activity is not exerted by the proteinaceous part of the Lpls but is rather due to cellular release of other PAMPs from the *lgt* mutant. The latter results from cellular stress induced by membrane jamming due to overexpression of the plasmid-encoded Lpls that cannot be correctly targeted to the membrane.

### Purified lipidated Lpl1-his—but not unlipidated Lpl1-his—induces proinflammatory cytokines in human-derived Mono Mac 6 and TLR2-transfected HEK293 cells

**Fig 6:**
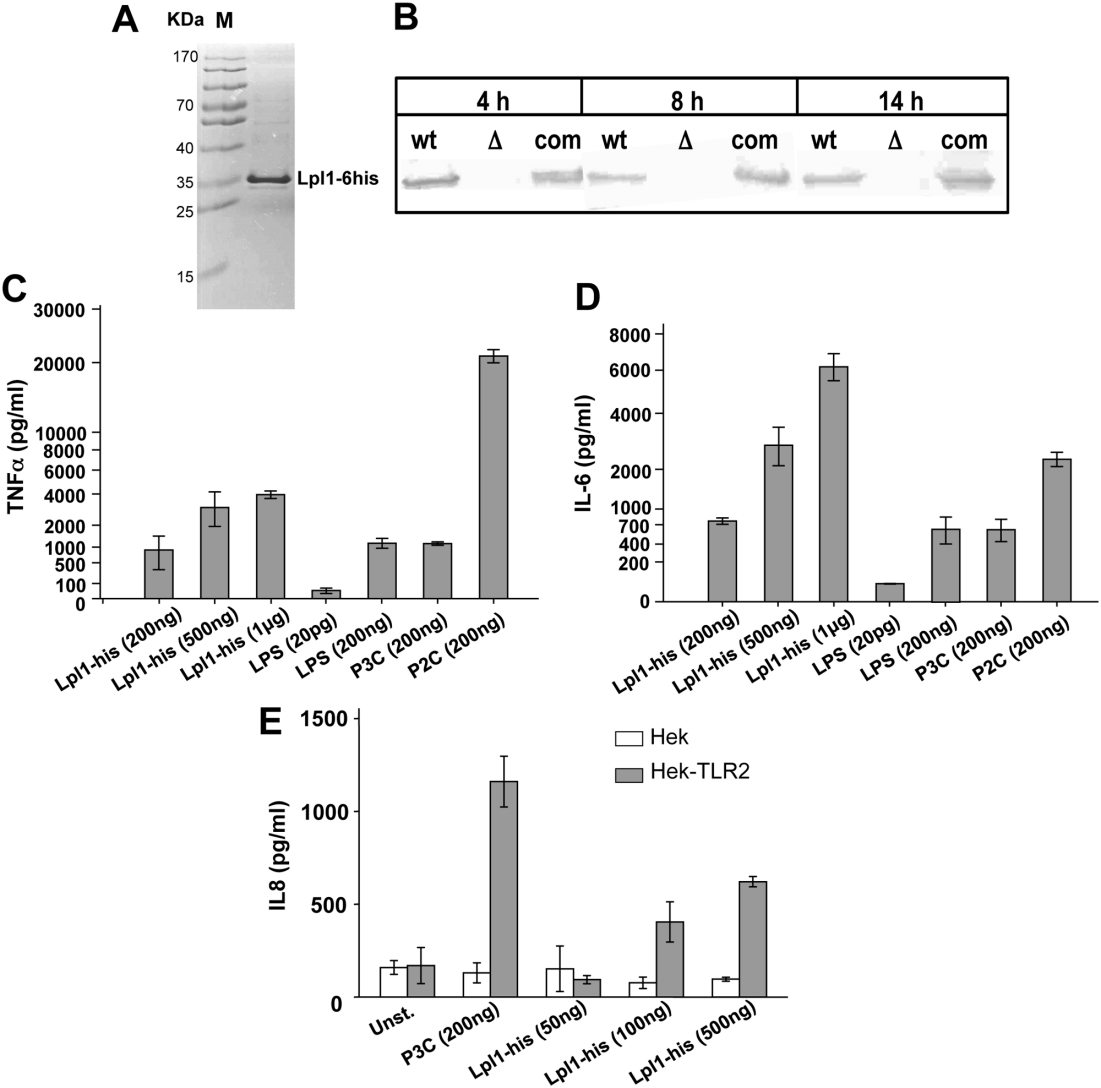
Induction of proinflammatory cytokines by purified Lpl1-his. (A) SDS-PAGE with purified Lpl1-his. (B) Western blots of antiLpl1-his with membrane fractions from USA300, its Δ*lpl* mutant and the complemented mutant (com). 10^6^ Mono Mac 6 cells were infected with different amounts (200 ng, 500 ng and 1 μg) of purified Lpl1-his. TNF-α (C) and IL-6 (D) were determined after 4 h and 24 h, respectively, of stimulation. Negative control was buffer with 20 pg of LPS; positive controls were each 200 ng of LPS, P3C and P2C. (**E**) HEK293 cells were transfected with or without a plasmid bearing TLR2 cDNA. Cells were stimulated with P3C (200 ng/ml), Lpl1-his (at the concentrations indicated in the diagram) or left unstimulated. IL-8 levels were quantified in cellular supernatants harvested after 24 h. One representative experiment performed in triplicates is shown. Error bars indicate standard error.

We, next, investigated the effect of a purified Lpl protein on the induction of cytokine secretion. As a prototype we chose the first *lpl*-gene (*lpl1*; 0410) of the *lpl* cluster, which also displays a high degree of similarity with the other four *lpl* genes (Fig 2). The *lpl1* was marked by a 3'- His-tag and was cloned into the pTX30::*lpl1*-his (Fig 1E). The Lpl1-his protein containing the lipid moiety (+sp) was purified from the membrane fraction of SA113 (pTX30::*lpl1*-his) by affinity chromatography and rabbits were immunized to generate anti-Lpl1-his rabbit antibodies. The purified Lpl1-his showed a high purity in Coomassie-stained SDS-PAGE (Fig 6A). In Western blot analysis of membrane fractions from USA300 wt, its Δ*lpl* mutant and the complemented mutant, Lpl1-his was only detectable in the wt and complemented mutant (Fig 6B). Notably, Lpl1-his expression did not undergo relevant changes after 4, 8 and 14 h cultivation periods.

Subsequently, purified Lpl1-his was used in concentrations of 200 ng, 500 ng and 1 μg/ml to stimulate Mono Mac 6 cells. P3C (200 ng), P2C (200 ng) and LPS (200 ng) were used as controls. The results showed that Lpl1-his induced TNF-α and IL-6 production in a concentration-dependent manner (Fig 6C and 6D). 200 ng Lpl1-his induced comparable amounts of TNF-α and IL-6 as seen with 200 ng of LPS or P3C. Surprisingly, we observed an extremely high stimulation of TNF-α by P2C, which was approximately 20 fold higher than that induced with the same amount of P3C or LPS. To further assess its immunogenicity purified Lpl1 was tested for TLR2 activity. HEK293 cells transfected with or without TLR2-bearing plasmid were stimulated with Lpl1 or TLR2 agonist P3C. Evidently, Lpl1-his induced IL-8 via TLR2 activation as a dose-dependent manner only was seen in the presence of TLR2 (Fig 6E). Of note, purified lipidated Lpl1-his was essentially endotoxin-free. Specificity of the assay was previously demonstrated by comparing SA113 and the SA113Δ*lgt* mutant that lacks TLR2-stimulating lipoproteins [1,18].

We also purified unlipidated Lpl1-his by expressing it in the absence of the lipo signal sequence (-sp) in *S*. *aureus* (Fig 1E). Purified unlipidated Lpl1 showed no stimulatory activity in Mono Mac 6 cells (S5 Fig), indicating that the lipidation of the Lpl proteins is essential for signaling, and the signaling observed in SA113Δ*lgt* (pTX30::*lpl*) was most likely due to stress-induced secondary PAMPs.

### The νSaα specific *lpl* cluster contributes to invasion into human keratinocytes and in mouse skin

**Fig 7:**
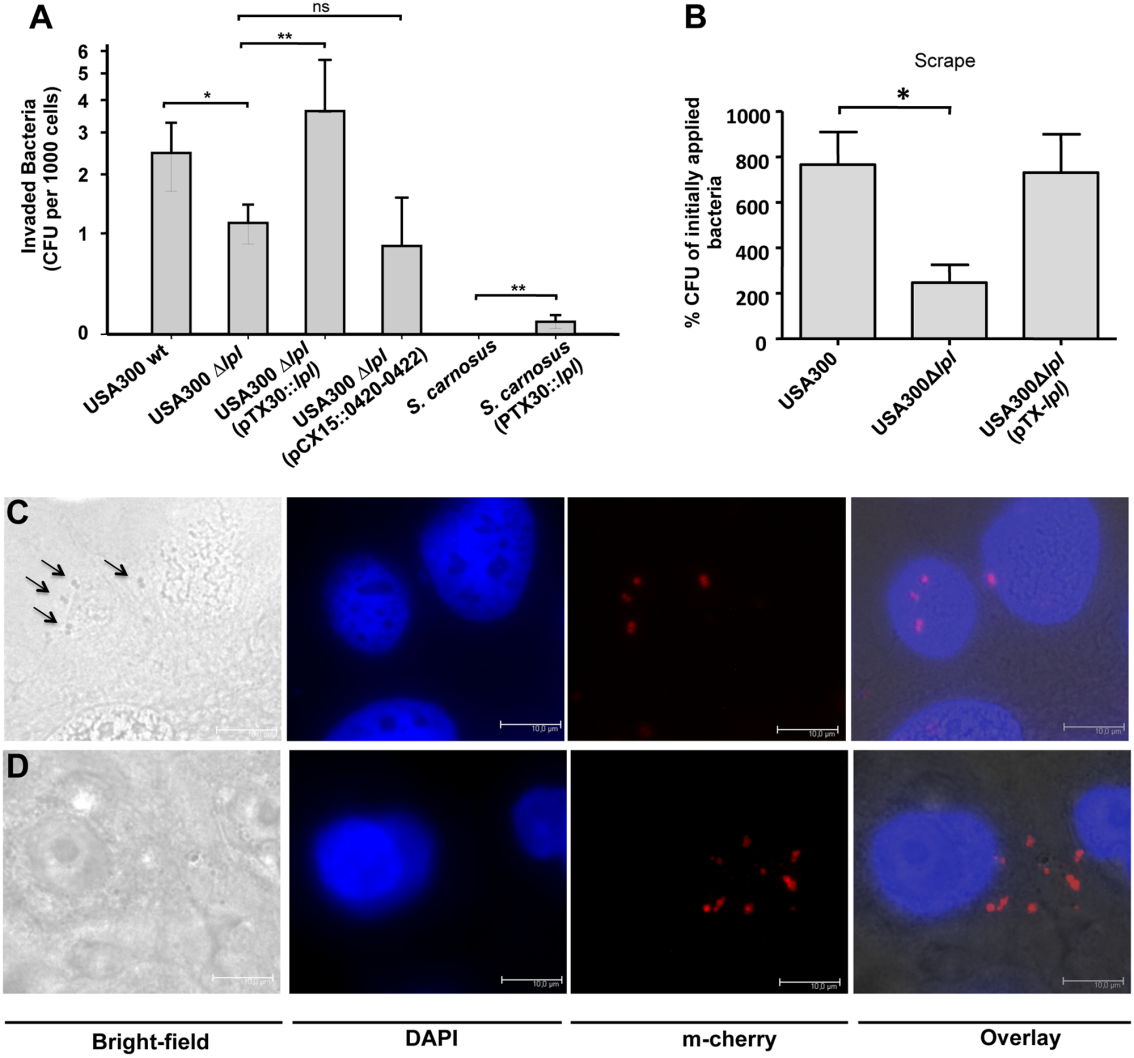
Invasion studies with human keratinocytes and mouse skin. (A) Differentiated primary human keratinocytes were infected with USA300, its Δ*lpl* mutant, the complemented mutant Δ*lpl* (pTX30::*lpl*), *S*. *carnosus* and *S*. *carnosus* (pTX30::*lpl*) with a MOI of 30. Infection was performed for 1.5 h, followed by additional 1.5 h antibiotic treatment. Two experiments performed in quadruplicates were conducted; at least 6 single values were used for statistical analysis by using analysis of variance (ANOVA). (B) Mid-logarithmic *S*. *aureus* cells were applied epicutaneously onto mouse skin, after shaving and tape-stripping (n = 6 mice). Infection occurred for 24 h, covered with Finn Chambers. For determination of invaded bacteria skin samples were resuspended in PBS. Several dilutions were plated on TSB agar plates. Statistical analysis was performed using ANOVA. Error bars indicate standard error of the mean (SEM). Statistical significances: not significant P>0.05; * P<0.05; ** P<0.01. Fluorescent microscopy images show the adherence (C) and invasion (D) of keratinocytes with mCherry expressing USA300. The images were automatically generated by using different filters for bright-field, DAPI and mCherry. DAPI images were used for detecting the nuclei of keratinocytes, and m-cherry images were used for detecting the USA300 bacteria; scale bar: 10 μm.

For invasion studies differentiated primary human keratinocytes were infected with USA300 wt, its Δ*lpl* mutant and the complemented mutant with a MOI of 30. In the Δ*lpl* mutant the number of invaded bacteria was 2.5-fold lower compared to the wt; in the complemented mutant the invaded bacteria exceeded that of the wt by 1.5-fold (Fig 7A). This suggests that the *lpl* gene cluster significantly contributes to invasion of keratinocytes. Downstream of the *lpl* cluster there are three (black-labeled) genes 0420–0422 with unknown function (Fig 1A). To exclude that these genes affect invasion we transferred the genes into USA300Δ*lpl* mutant by pCX15::0420–0422. The results confirmed that these genes do not enhance invasion (Fig 7A). We further investigated the invasion of *S*. *carnosus* and *S*. *carnosus* (pTX30::*lpl*). While *S*. *carnosus* and *S*. *carnosus* (pTX30) completely lacked invasion, *S*. *carnosus* (pTX30::*lpl*) showed a significant increase in invasive activity, which corresponds to approximately 5% of that seen with USA300 (Fig 7A). Thus, these results provided strong evidence that the Lpl proteins trigger host cell invasion.

To clarify whether invasion can also be observed *in vivo*, we, next, carried out invasion studies on murine skin after shaving and tape-stripping (Fig 7B). Here, too, we confirmed significantly lower invasion with the mutant compared to the wt; and in the complemented mutant invasion could be restored to the extent reached by the wt. In order to demonstrate that USA300 is internalized into human keratinocytes we monitored bacterial adherence and invasion with a mCherry-expressing USA300 strain. Using bright-field microscopy we could visualize adherence of USA300 to the host cell surface (Fig 7C). In the invasion studies adherent USA300 cells were lysed with lysostaphine. We could, therefore, not detect them in the bright-field microscopy but only by fluorescence microscopy (Fig 7D).

### The νSaα-specific *lpl* cluster enhances the bacterial burden in a mouse kidney abscess model

**Fig 8:**
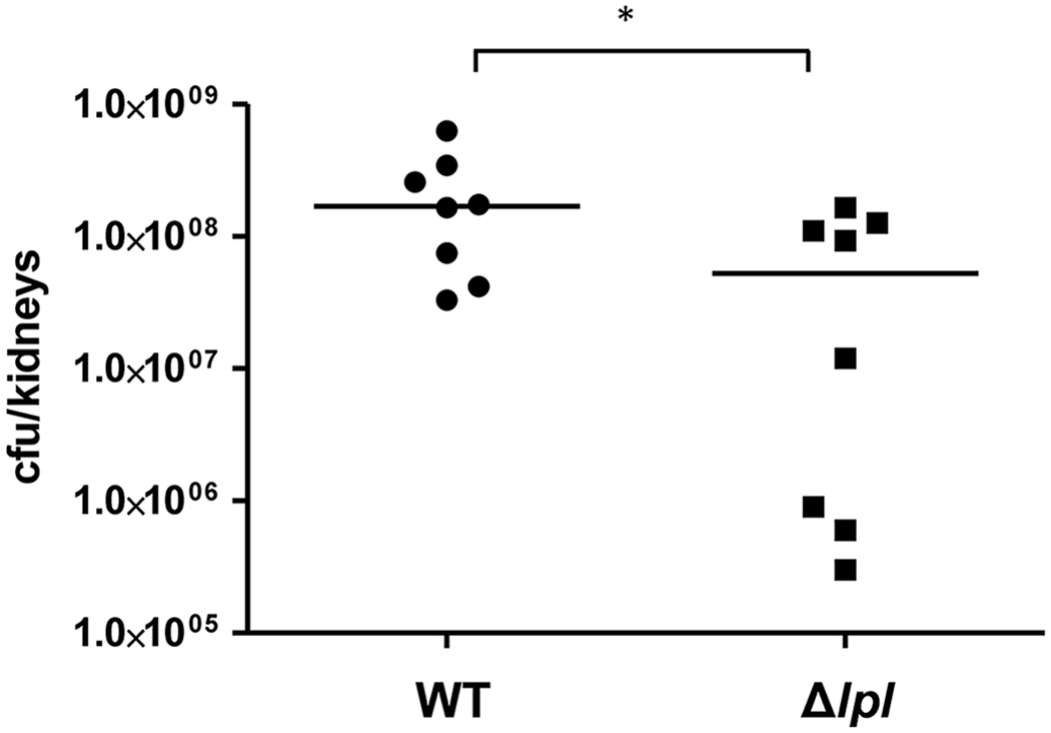
Mouse kidney abscess model. Balb/c mice were infected with 5x10^6^ CFU of USA300 (n = 8 mice), or its Δ*lpl* mutant (n = 8 mice). Kidneys were removed and homogenized at day 5 post-infection and bacterial burden determined through viable count (CFU/kidneys). Horizontal line represents median log10 CFU/kidneys; significant differences (* P<0.05) in bacterial burden were noted between parent strain and its Δ*lpl* mutant. Data were analyzed using Mann-Whitney test.

To see whether the *lpl* cluster contributes to pathogenicity we examined USA300 and the *Δlpl* mutant in a mouse kidney abscess model with Balb/c mice infected for 5 days. With the *Δlpl* mutant the bacterial burden of the kidneys was significantly lower when compared to the parental strain (Fig 8). The complemented mutant Δ*lpl* (pTX30::*lpl*) was also tested in the kidney abscess model, however, the number of colony forming units (CFU) did not reach the level of the parental strain. We assume that the xylose-inducible expression of the *lpl* genes was instable over the 5-day infection period. Additionally, we observed a slight growth defect in TSB with clones containing the high-copy plasmid pTX30.

## Discussion

Why the genomic island νSaα is so prevalent and highly conserved in *S*. *aureus* is a puzzling issue. For the genetic maintenance of the tandem *lpl* gene cluster it is currently believed that the highly conserved intragenic region flanked by the variable regions of the *lpl* genes is responsible for gene shuffling, tandem duplications and diversification [7]. On the other hand, if νSaα was not beneficial in infection we would expect it to get lost during evolution. Thus, the aim of this study was to investigate the potential benefit of the νSaα tandem *lpl* gene cluster in infection.

We chose *S*. *aureus* USA300 [9] as a model strain for several reasons: firstly it contains one of the most complex tandem lipoprotein clusters in the νSAα island, and, secondly, it is a member of the clonal complex (CC) 8 that encompasses several globally distributed epidemic lineages, including hospital-associated methicillin-resistant *S*. *aureus* (MRSA) and the highly prevalent community-associated MRSA clone USA300 [19]. However, the genetic basis for the high transmissibility and hypervirulence of the CC8 strains, and USA300 in particular, is unknown. Here, we reasoned that the νSaα island could represent a virulence determinant of *S*. *aureus*. After some difficulties we managed to construct a marker-less deletion mutant of the 10 kb *lpl* cluster. The mutant showed no apparent growth defect in both B-medium (rich medium) and DMEM/F (nutrient-poor medium) indicating that the tandem lipoproteins have no vital role *in vitro*, however, they might contribute to innate immune response.

It is well known that lipoproteins and lipopeptides are recognized by TLR2 [20,21] which can form heterodimers with TLR1 or TLR6 to trigger intracellular signaling by triacylated and diacylated lipopeptides, respectively [22–25]. In *Staphylococcus* lipoproteins do not only contribute to ion uptake and pathogenicity, they also represent the predominant immune stimulators [1,6,26–28]. Therefore, we first addressed whether and to which extent the *lpl* gene cluster contributes to innate immune stimulation. Searching the annotated proteins of USA300 by the DOLOP program there are approximately 63 proposed lipoproteins present including the 10 Lpl proteins of the νSaα island. The 10 Lpl proteins comprise 16% of all lipoproteins in USA300. Theoretically, one would expect only a minor effect on induction of pro-inflammatory cytokine expression in Mono Mac 6 cells. However, we observed in USA300Δ*lpl* a 3-fold decrease in TNF-α and an almost 2-fold decrease in IL-6 expression (Fig 3A and 3C). The complemented strain USA300*lpl* (pTX30::*lpl*) largely reached parental expression levels even in the absence of xylose, while in the presence of xylose TNF-α and IL-6 expression exceeded that of the parental strain by a factor of 2 and 4, respectively (Fig 3A and 3C). These results show that despite the presence of other lipoproteins Lpl proteins have an unexpectedly high impact on innate immune stimulation. Notably, heterologous expression of pTX30::*lpl* in *S*. *aureus* HG003 caused a similarly high induction of TNF-α and IL-6 expression as with USA300 (Fig 4A and 4B).

Of note, in *S*. *carnosus* the induction of TNF-α and IL-6 expression was not as pronounced. However, the non-pathogenic *S*. *carnosus* was exceptional in so far, as it triggered an almost 20-fold higher TNF-α and IL-6 expression than the *S*. *aureus* strains. *S*. *carnosus* belongs to the non-pathogenic staphylococcal species and we assume that generally the non-pathogenic species are non-pathogenic not only because of low to absent toxin and adhesion production but also because of their better recognition by our immune system [17]. Interestingly, we also observed a comparable difference in stimulation with the synthetic lipopeptides: P2C was much more active than P3C (Fig 6C).

Since in *S*. *aureus* lipidation of the lipoproteins by diacylglyceryl transferase (encoded by the *lgt* gene) is crucial for innate immune activation [1], we wondered whether innate immune stimulation is preserved after cloning of the *lpl* gene cluster into an *lgt* deletion mutant. As shown in Fig 4C and 4D there was a roughly 2-fold increase in TNF-α and IL-6 expression in SA113*lgt* (pTX30::*lpl*) compared to SA113*lgt*. Although SA113*lgt* (pTX30::*lpl*) did not reach the SA113 parental level, we wondered why there was an increase at all. The most likely explanation is release of other PAMPs as a stress-response due to the *lgt* mutation. In this mutant all pro-lipoproteins are unlipidated but they are still transiently anchored in the cytoplasmic membrane by the unprocessed signal peptide. Taking into consideration that in SA113*lgt* (pTX30::*lpl*) ten additional lipoproteins are highly expressed and cannot be correctly targeted via their lipid moiety to the outer leaflet of the cytoplasmic membrane, the membrane translocation machinery might be crammed, which could result in release of peptidoglycan and/or DNA that could contribute to the observed innate immune stimulation. Membrane jamming has frequently been observed when proteins were overexpressed and not correctly targeted. For example, upon overproduction of β-galactosidase hybrid proteins, the export pathway is so severely jammed that other exported proteins accumulate in their precursor form in the cytoplasm [29]. The other thrilling but unlikely possibility would have been that unlipidated Lpls caused immune stimulation. To clarify the situation we purified his-tagged Lpl1, as a Lpl representative lipoprotein, with and without its lipid part by expressing it with and without its lipo signal sequence (Fig 1E) in *S*. *aureus*. Although applied in high concentration, the unlipidated protein Lpl1 showed no immune stimulating activity, suggesting that the increased immune stimulating activity in SA113*lgt* (pTX30::*lpl*) is probably due to a side effect, possibly, by higher release of peptidoglycan fragments, DNA or RNA that also contribute to innate immune stimulation [30]. Lastly, Lpl-mediated invasive activity might facilitate uptake and cytosolic sensing of these bacterial compounds.

The enhanced innate immune stimulation exerted by the *lpl* gene cluster in USA300 is not really beneficial in regards to virulence; on the contrary, these strains are better recognized by the innate immune system. Therefore, the key question was whether expression of the νSaα tandem *lpl* gene cluster could represent an advantage in infection. It turned out that the most beneficial function of the Lpl was increased invasiveness. Invasion studies *in vitro* and *in vivo* (Fig 7A and 7B) revealed that the mutant USA300Δ*lpl* was significantly less invasive (>2-fold) than the parental strain, and that the complemented mutant showed an even higher invasiveness than the parental strain. The ability of *S*. *aureus* to be internalized by host cells is considered to be one of the most critical pathogenicity factors in persisting and relapsing infections because intracellular localization of bacteria evades antimicrobials and the host immune response [31]. It is now widely accepted that *S*. *aureus* is internalized by a variety of non-professional phagocytes, such as endothelial cells [32,33], epithelial cells [34–36], fibroblasts [37], or osteoblasts [38,39], and can persist inside the host cells for weeks, months or even years. The fibronectin binding proteins (FnBPA and FnBPB) are the major adhesins involved in *S*. *aureus* internalization by host cells. They use fibronectin as a bridging molecule and the α_5_β_1_ integrin as host cell receptor resulting in signal transduction, tyrosine kinase activity, and cytoskeletal rearrangements [34,36,40]. The FnBPs also bind to the heat shock protein 60 (Hsp60), which might act as a co-receptor in the FnBP-mediated uptake of *S*. *aureus* [41]. Furthermore, a role of the extracellular adherence protein (Eap) was described in internalization into fibroblasts or epithelial cells; the receptor is unknown to date [42]. Recently, the major autolysin of *S*. *aureus* and *S*. *epidermidis* Atl was found to contribute to host cell internalization by interacting with the heat shock cognate protein Hsc70 [43].

A very important confirmation of the invasive activity of the Lpls was the finding that the non-pathogenic and non-invasive *S*. *carnosus* strain becomes invasive when transformed with (pTX30::*lpl*) (Fig 7A). Although the frequency of invasion of S. *carnosus* (pTX30::*lpl*) was low, e.g. only approximately 10% of that of USA300, one has to consider that *S*. *carnosus* lacks FnBPs as the major players of *S*. *aureus* invasion and also Eap [44]. Furthermore, we do not know whether Atl plays a role in *S*. *carnosus*-mediated host cell invasion as its sequence and domain organization differs significantly from that of *S*. *aureus* [45]. Therefore, the invasive activity seen in *S*. *carnosus* probably reflects the impact of the Lpls.

In our studies we frequently used human keratinocytes because they represent the immune sentinels in the skin and as such they provide a first line of defense against microbial pathogens. They express almost all TLRs, which is crucial for promoting skin immune responses to adherent bacteria. Activation of these receptors on human keratinocytes leads to a predominant TH1-type immune response and to the production of type I interferons (IFNs) [46]. Moreover, keratinocytes also constitutively secrete, or are induced to release, numerous cytokines, including IL-1, IL-6, IL-10, IL-18 and TNF-α; and they process and release IL-1β through activation of the inflammasome.

Increased invasive activity is an important advantage for persistence. In our opinion, this overrules the disadvantage of enhanced innate immune stimulation. It is well known that if a pathogen invades new tissues the host responds by eliciting immune responses in an effort to eliminate infection. Internalization into non-professional antigen-presenting cells such as keratinocytes [46] most likely protects *S*. *aureus* from the immune response. It is, therefore, to expect that by contributing to host cell invasion the *lpl* gene cluster enhances *S*. *aureus*' virulence and particularly its persistence. Whether the enhanced host cell invasion also contributes to increased dissemination and epidemic spreading needs to be further investigated.

*Lpl* gene clusters are found in most *S*. *aureus* strains; however, these clusters differ with respect to the number of the tandem *lpl* genes and the type of νSaα. For example, most members of the CC8 (USA300, Newman, A5948, A9754) have 10 *lpl* genes, while NCTC8325 has only three. Members of the CC5 lineage also have an average of 10 *lpl* genes. Both CC5 and CC8 are distinguished by type I νSaα, while CC1, CC30 and CC151 possess type II, III and IV, respectively (S6 Fig). In this study we chose USA300 as a model strain for *lpl* gene cluster analysis. However, the *lpl* gene cluster in other *S*. *aureus* strains will most likely have a similar function in invasion. One of the questions which will be addressed in the future is whether the number of *lpl* repeats in νSaα is correlated with the invasion frequency. Probably, there is a fine-tuned balance between a stable maintenance of the repeats and their benefit in infection. We further performed electron microscopy to visualize potential differences in cell surface structures between USA300 and its Δ*lpl* mutant. However, at first sight these images revealed no apparent differences in the morphology (S7 Fig). This was not unexpected because the mutant expresses 50 other lipoproteins.

Recently, it has been shown that *S*. *aureus* USA300 and HG001 induced autophagy in a human tumor cell line, while *S*. *carnosus* did not [47]. The *S*. *aureus* cells become entrapped in vesicles and dividing *S*. *aureus* cells were seen intracellularly suggesting that *S*. *aureus* cells are able to multiply intracellularly [47]. Whether the *lpl* gene cluster is involved in this process is currently unknown. However, the ability of *S*. *aureus* to invade different types of non-professional phagocytes, to escape from the host lysosomal degradation machinery and to persist within the intracellular location most likely represent essential steps in pathogenesis. After invasion there are several options: *S*. *aureus* cells may be killed by the host cell, they kill the host cell, or they survive and persist by switching to an SCV (small colony variant) phenotype, an essential step for establishing chronic infections [48].

What distinguishes an epidemic and disseminating strain from other strains is an important question. Not many cases are known where epidemic outbreaks could be traced to certain genes. One recent example is the *Escherichia coli* O104:H4 strain that caused the large German outbreak in 2011. This strain acquired genes from an entero-aggregative *E*. *coli* strain (EAEC) that increased intestinal colonization thus turning it into a highly virulent and disseminating hybrid strain [49,50]. In *Listeria monocytogenes* the interaction between the bacterial surface molecules, the internalins InlA and InlB, and their cellular receptors E-cadherin and the Hepatocyte Growth Factor Receptor (Met), respectively, triggers the recruitment of endocytic effectors, the subversion of the phosphoinositide metabolism, and the remodeling of the actin cytoskeleton that leads to bacterial engulfment [51]. In this pathogen the internalins are the basic virulence factors because they are a prerequisite for spreading and dissemination. It is, however, completely unknown how the Lpl proteins trigger invasion.

### Conclusion

This is one of the first studies addressing the role of the νSaα-encoded tandem *lpl* gene cluster in a member of the high transmissible and hypervirulent clonal complex CC8. Firstly, the *lpl* gene cluster enhances the innate immune signaling suggesting that the lipoproteins carry the lipid moiety, which has been confirmed by testing purified lipidated and unlipidated Lpl1 lipoprotein *in vitro*. On the one hand, increased activation of the innate immune system is not really beneficial for a pathogen because it activates bacterial immune defense. On the other hand, however, it is highly beneficial for a first-class pathogen to acquire an enhanced ability to invade host cells, which may facilitate spreading in the host. This is illustrated by the enhanced bacterial burden in a murine kidney abscess model. The increased ability to invade host cells could further be responsible for the high disseminative activity of some *S*. *aure*us isolates. Investigation of the exact mechanism of host cell invasion is one of the expedient next steps. We postulate that the *lpl* gene cluster will have clinical, diagnostic, epidemiologic and evolutionary implications.

## Materials and Methods

### Bacterial strains and growth conditions

Bacterial strains and plasmids used in this study are listed in S1 Table. *E*. *coli* strains were cultivated aerobically in Luria Broth (LB) medium with shacking at 30°C. *S*. *aureus* strains aerobically grown in either basic medium, BM (1% soy peptone, 0.5% yeast extract, 0.5% NaCl, 0.1% glucose and 0.1% K_2_HPO_4_, pH 7.4), or Tryptic Soy Broth (TSB) at 37°C. When appropriate, the media were supplemented with ampicillin (100 μg/ml), erythromycin (10 μg/ml), tetracycline (25 μg/ml) or chloramphenicol (20 μg/ml).

### Deletion of the tandem lipoprotein cluster by allelic replacement in *S*. *aureus* USA300

The deletion of entire *lpl* cluster in *S*. *aureus* USA300 was generated by homologous recombination. The basis for the construction of the knock-out plasmid was temperature-sensitive *E*. *coli—Staphylococcus* shuttle vector pBT2 [52] in which three DNA fragments were cloned between the EcoRI and BamHI sites: the approximately 1 kb upstream region comprised part of ORF 0408 and the entire ORF 0409, the 1 kb downstream region comprised ORF 0423 and part of ORF 0424. In between the up- and downstream region the 1.2 kb erythromycin gene flanked by *loxP* sites was integrated (Fig 1A). The 1 kb upstream and downstream regions were amplified by PCR from the genome of *S*. *aureus* USA300 using primer pairs Fr_up (EcoRI) and Re_up (Xhol) and primer pair Fr_down (XbaI) and Re_down (BamHI), respectively (S2 Table). The *ermB* gene together with the flanking *loxP* sites was amplified from pBT2-srtA [53] with primer pair Fr_*erm* (XbaI) and Re_*erm* (XhoI); the Cre*-loxP* sequence is in italics. The amplified upstream fragment was cut with EcoRI and XhoI, the amplified downstream fragment was cut with XbaI and BamHI, and the amplified *ermB* gene fragment was cut with XhoI and XbaI. These fragments were then ligated with vector pBT2 digested by BamHI and EcoRI, resulting pBT2Δ*lpl* and transformed into *E*. *coli* DH5α. The positive clone containing plasmid pBT2Δ*lpl* was isolated, purified and subsequently transformed by electroporation into *S*. *aureus* RN4220 as an intermediary host and then transformed into *S*. *aureus* USA300. The deletion of tandem lipoprotein cluster from genome was achieved by homologous recombination described previously [52]. The resultant deletion mutant was named USA300Δ*lpl*::*erm*B (Fig 1A). This clone was transformed with thermo-sensitive replicon pRAB1 which encodes the *cre*-recombinase gene to remove the *erm*B cassette [53]. Positive clones (erythromycin and chloramphenicol sensitive) were selected and confirmed by PCR and sequencing (GATC-Biotech AG, Konstanz, Germany).

### Construction of pTX30::*lpl* and pCX15::0420–0422

Plasmid pTX30::*lpl* contained all ten *lpl* genes under control of the xylose-inducible promoter. pTX30 is a derivative of pTX15 [54] with a strong transcription terminator inserted and a SalI site deleted by partial digestion. Into pTX30 the entire *lpl* gene cluster (10,529 bp) was inserted stepwise. The first fragment (4,146 bp) comprising the first five lipoprotein genes (USA300 0410–0414) was amplified by using forward primer F_0410 (BamHI) and reversed primer Re_0414 (Sal1). The second fragment (3,537 bp) comprising the 4 next lipoprotein genes (0415–0418) was amplified by using forward primer F_0415 (Sal1) and reversed primer Re 0418 (AvrII). The last fragment (2,892 bp) containing the last lipoprotein gene (USA300-0419) together with the three *lpl* flanking genes (USA300 0420–0422) were amplified by using forward primer F_0419 (AvrII) and reversed primer R_0422 (SacI). The amplified fragments and plasmid pTX30-mch-cw were cut by using the same digestion enzymes and subsequently ligated together. The ligation products were transformed first into *S*. *carnosus* TM300 by the protoplasts method [55] to yield the plasmid pTX30::*lpl* (17,343 bp), which was then transformed into *S*. *aureus* RN4220 and USA300Δ*lpl*.

Plasmid pCX15::0420–0422 contained three genes (SAUSA 0420–0422) which are located at the end of the *lpl* cluster and are highly conserved. The corresponding DNA fragment (2,020 bp) was amplified by using two primers Fr_0420 and Re_0422 and were inserted between the BamHI and XbaI site of pCX15 [56]. The ligation products were first transformed into *S*. *aureus* RN4220 by electroporation; the resulting plasmid, pCX15::0420–0422 (5,912 bp), verified by DNA sequencing was subsequently transformed into *S*. *aureus* USA300Δ*lpl* and *S*. *carnosus* TM300.

### Construction of pTX30::*lpl1*-his with and without signal sequence

A fragment containing the first *lpl* gene SAUSA0410, named Lpl1-his, was amplified from the USA300 genome by using primers containing 6xHis-tag codons at the 3' end. We constructed two different lipoproteins Lpl1-his with and without signal sequence. The forward primers of both construction comprises an optimized SD-sequence AGGAGG, downstream a BamHI site, and upstream with the start codon and following the complemented sequence (S2 Table). Two constructed plasmid containing the same reversed primer, Re_Lpl1-his (SacI), comprises a 6-Histag coding sequence (sequence in italics), a stop codon and a SacI site. The amplified fragment was ligated into xylose inducible vector pTX30 after digestion with two BamHI and SacI enzymes to yield the plasmid pTX30::*lpl1*-his. This plasmid was transformed into *S*. *aureus* SA113 by electroporation.

### Purification of Lpl1-his

Lpl1-his was isolated from the membrane fraction of *S*. *aureus* SA113 (pTX30::*lpl1*-his). The clone was first cultivated aerobically at 37°C in the absence of xylose (BO-medium) until OD_578nm_ ≈ 0.5 was reached, then 0.5% xylose was added to induce Lpl1 expression and cultivation was continued for 4 h. The bacterial cells were harvested by centrifugation at 4,000 x g at 4°C. The cell pellets were washed two times with Tris buffer (20 mM Tris, 100 mM HCl, pH 8.0). Then the pellet was re-suspended with Tris buffer containing protease inhibitor table (Merck, Darmstadt, Germany) and lysostaphin (30 μg/ml) and incubated at 37°C for 2 h to disrupt the cell wall. Membrane protein extraction and purification were followed according to a previous study [27] with a small modification. After ultracentrifugation (235,000 x g for 45 min at 4°C), membrane proteins were dissolved overnight at 6°C with Tris buffer containing 2% Triton X100. After another ultracentrifugation step, the supernatant was incubated with Ni-NTA super flow beads (Qiagen, Germany) overnight at 6°C under mild rotation 20 rpm. One volume of Ni-NTA beads were washed twice with five volumes of extraction buffer (Tris buffer containing 0.25% TritonX-100 and 20 mM imidazole), subsequently the beads were washed four times with five volumes of the same buffer containing 40 mM imidazole and finally the Lpl1-his was eluted with the same buffer containing 500 mM imidazole. Lpl1-his was concentrated via centrifugal ultra-filter unit with a molecular mass cut-off of 10 kDa (Sartorius AG, Göttingen, Germany). Finally, the Lpl1-his purification was checked by SDS-PAGE and the total protein amount was determined by a Bradford assay kit. Purified Lpl1-his was used for generating polyclonal rabbit antibodies by Biogenes GmbH (Berlin, Germany).

The purification of Lpl1-his (-sp) was performed with the same procedures as Lpl1-his (+sp) except that the Lpl1-his (-sp) isolated from the cytoplasm of *S*. *aureus* SA113 by French press.

### Determination of endotoxin contamination

The purified lipoprotein (Lpl1-his) stock used for stimulation of cytokine production was tested for endotoxin contamination as 0.19 EU (1 EU≅100 pg LPS) by the Endosafe-PTS system (Charles River, Charleston, USA). Positive reaction is usually due to LPS contamination but might also arise from lipoprotein content.

### Localization of Lpl1-his

USA300 was cultivated aerobically in B-medium and cells were harvested after 4, 8 and 14 h. The cells were harvested by centrifugation (4000 x g, for 10 min at 4°C) and the supernatant was filtered with 0.2 μl Millipore filters to remove remaining cells. Two ml of supernatant were mixed with 10 μl StrataClean Resin (Agilent Technology, USA) and incubated for 5 min at room temperature. After centrifugation (12,000 x g, for 10 min, at 20°C), the pelleted resin with bound extracellular proteins was washed twice with 20 mM Tris buffer (pH 8). The membrane fraction was isolated as described above and membrane proteins were precipitated using chloroform and methanol method [57]. The extracellular and membrane proteins were dissolved in SDS running buffer, separated by SDS-PAGE and transferred onto nitrocellulose membrane by using semi-dry transfer apparatus in semidry buffer (25 mM Tris, 150 mM glycine, and 10% methanol) using 350 mA for 80 min. Lpl1-his was detected by Western blot using anti-Lpl1-his rabbit antibodies at a dilution of 1:1000.

### Human cells and infection assay

Mono Mac 6 [58], a human monocytic leukemia cell line, was obtained from DSMZ (Braunschweig, Germany) and cultured in RPMI-1640 (Biochrom AG, Berlin, Germany) supplemented with 10% FBS superior (BiochromAG, Berlin, Germany), 1% OPI (O5003, Sigma, Taufkirchen, Germany) in NEA-non essential amino acids NEA (Biochrom AG, Berlin, Germany) and 1% Zell Shield (Minerva Biolabs GmbH, Berlin, Germany) at 37°C with 5% CO_2_. Prior to stimulation, 10^6^ cells per 24-well microtiter plate were seeded out in 1ml of culture medium and incubated for 1 h at 37°C with 5% CO_2_ supplement. Mono Mac 6 cells were stimulated with staphylococcal strains with ratio MOI 30:1 or otherwise specifically indicated. Prior to stimulation, staphylococcal strains were cultured overnight in TSB or TSB with 0.8% xylose and washed twice with Dulbecco’s Modified Eagle Medium (DMEM/F-12) containing L-glutamine and 15mM HEPES (Gibco). The time period of stimulation was 4 h for TNF-α or 24 h for IL-6. The supernatants were collected and stored at -20°C until usage. In addition, different amounts of Lpl1-his (200 ng, 500 ng and 1 μg) were applied to 10^6^ Mono Mac 6 cells and their supernatants were collected after 4 h and 24 h incubation for TNF-α and IL-6 measurement, respectively. LPS (Sigma Aldrich, Taufkirchen, Germany), Pam3Cys (P3C), and Pam2Cys (P2C) (EMC, Tuebingen, Germany) were used as controls.

Peripheral human blood mononuclear cells (PBMC) were isolated from buffy coats by Ficoll gradient centrifugation. Culture medium consisting of RPMI 1640 (PAA, Vienna, Austria) supplemented with 1% L-glutamine and 1% penicillin-streptomycin (both from Sigma-Aldrich, Munich, Germany) and 10% FCS (PAA Laboratories, Vienna, Austria) or 2% human antibody positive serum (Lonza, Cologne, Germany) for macrophage cultures. Medium supplements were controlled for endotoxin contamination by absence of TNF induction [59]. Human macrophages were obtained by culturing the adherent cell fraction from PBMC for five days in the presence of GM-CSF (10 ng/ml on day 0 and day 3, Miltenyi Biotec, Bergisch-Gladbach, Germany) or M-CSF (10 ng/ml on day 0 and day 3; Miltenyi Biotec). Cells were stimulated in 24-well plates for 24 hours with bacteria (MOI 10:1) or left unstimulated.

Transfection of adherent HEK293 cells 5x10^4^ per well (200 μL) was performed with lipofectamine (Invitrogen, Karlsruhe, Germany) with or without 200 ng plasmid bearing TLR2 cDNA overnight. After washing, cells were stimulated with TLR2 ligands P3C (100 ng/ml), different amounts of Lpl1-his (50 ng, 100 ng and 500 μg) for 24 hours before harvest of cellular supernatants. TLR2 activity was assessed via quantification of secreted human IL-8 by ELISA (BD OptEIA, BD Biosciences, Heidelberg, Germany).

### Cytokine and antimicrobial peptide (AMP) expression in differentiated primary human keratinocytes

With *S*. *aureus* it is difficult to determine cytokine concentration by ELISA because of IgG-binding protein A that affects Western and ELISA assays. Therefore, we used frequently transcript analysis as has been carried out previously [60]. Primary keratinocytes were isolated from human foreskin after routine circumcision. Cultivation of the cells was performed in collagen-coated flasks in keratinocytes growth medium (KGM) at 37°C, 5% CO_2_ as described previously [61]. Prior to infection, the cells were seeded into collagen-coated 24-well plates. For differentiation, medium was changed to keratinocyte base medium (KBM; KGM lacking BPE, supplements and antibiotics) and calcium concentration of KBM medium (0.15 mM) was altered to 1.7 mM. The cells were infected for 8 h with *S*. *aureus* USA300 strains with a MOI of 30 (±10). Before stimulation, *S*. *aureus* strains (USA300 wild type, USA300Δ*lpl* and complemented mutant USA300Δ*lpl* (pTX30::*lpl*) were cultured overnight in TSB and washed two times with PBS. Bacterial pellets were resuspended in KBM without any supplements for treatment of the cells. RNA extraction (NucleoSpin RNA, Macherey-Nagel, Düren, Germany) and reverse transcription (SuperScript II Reverse Transcriptase, Invitrogen, Carlsbad, USA) were performed according to the manufacturer's instructions. Quantitative RT-PCR for measurement of gene expression was performed using KAPA SYBR Fast (Peqlab, Erlangen, Germany) with the Roche LightCycler 480 Real-Time PCR system. Relative expression of target genes was calculated as ratio to β-actin as reference gene. Primer sequences are listed in S3 Table.

### Detection of cytokines and immunoglobulin by ELISA

Human IL-8, TNF-α, IL-6 and IL-10 secretion was measured in cellular supernatants using the BD OptEIA ELISA kits according to the manufacturer´s instructions.

### Murine kidney abscess model

Female BALB/c mice (18–20 g) were purchased from Charles River, Sulzfeld, Germany, housed in polypropylene cages and supplied with food and water ad libitum. *S*. *aureus* were cultured for 18 h in B-medium, washed 3 times with sterile PBS and suspended in sterile PBS to the desired concentration. To verify viable cell counts, appropriate dilutions were plated on B agar plates. Mice were inoculated with 100 μl of *S*. *aureus* via the tail vein. Eight mice were used for each strain of *S*. *aureus* tested. Five days after bacterial challenge, the kidneys were aseptically harvested and used for CFU enumeration. Kidneys were homogenized in 3 ml of sterile PBS using Dispomix (Bio-Budget Technologies GmbH, Krefeld, Germany) and serial dilutions of the organ homogenates were cultured on mannitol salt-phenol red agar plates for at least 48 h at 37°C. Colony-forming units were counted and the bacterial burden was calculated as CFU/kidneys. The statistical significance was determined using the Mann–Whitney test for bacterial burden.

### Adherence and invasion of *S*. *aureus* USA300 in differentiated primary human keratinocytes and mouse skin

Differentiated primary human keratinocytes in 24-well plates were cultured as described above and were infected with USA300, its Δ*lpl* mutant and the complemented mutant Δ*lpl* (pTX30::*lpl*) with a MOI of 30. Before stimulation, the strains were cultured overnight in TSB and washed two times with PBS. Bacterial pellets were resuspended in KBM without any supplements and incubated with host cells for 1.5 h. For determination of the number of adhered bacteria, the cells were washed three times and lysed 1.5 h after infection and several dilutions were plated on TSB agar. For detection of invaded bacteria, cells were treated with lysostaphin (Sigma-Aldrich, Taufkirchen, Germany) for additional 1.5 h to remove extracellular bacteria. Keratinocytes were lysed with 0.1% Triton X-100 and 0.5% Trypsin in PBS. Agar plates were incubated overnight at 37°C and the obtained numbers of CFU were normalized to keratinocyte numbers.

For analysis of invasion in mouse skin, epicutaneous infection with *S*. *aureus* USA300 was performed. *S*. *aureus* strains (USA300, USA300Δ*tlpp* and USA300Δ*tlpp* (pTX30::*tlpp*) were cultured overnight in TSB. Afterwards they were transferred into pre-warmed TSB and grown to mid-logarithmic phase. Tape-stripping method is an established mouse model to follow the penetration of e.g. *S*. *aureus* into epidermal keratinocyte layers and the subsequent dissemination; the experimental infections do produce significant dermal damage, but the latter develops after dissemination has already taken place [62]. Here, C57BL/6 mice (Charles River Laboratories, Sulzfeld, Germany) were shaved and tape-stripping was performed 7 times (strong tape-stripping, STS). An inoculum of 3.5x10^6^ (±1.5x10^6^) bacteria was added to filter paper discs, placed onto the skin and covered by Finn Chambers on Scanpor (Smart Practice, Phoenix, USA). Overnight fixation occurred via Fixomull stretch plaster (BSN medical, Hamburg, Germany). 24 h after infection Finn Chambers and plasters were removed and two skin samples per mouse from the application site were taken. For determination of CFU skin samples were first washed in PBS to remove loosely attached bacteria and then scraped in 1 ml PBS to obtain the number of invaded bacteria. Several dilutions were plated on TSB agar plates and incubated overnight at 37°C. The number of invaded bacteria was calculated as percentage of initially applied CFU onto the skin sample.

### Fluorescent microscopy

Staphylococcal strains were transformed with pCtuf-pp-mch [47] prior to incubation with 10^5^ differentiated primary human keratinocyte cells in four-well chamber slides (Omnilab, Bremen, Germany). Adhered and invaded bacteria were microscopically analyzed. The cells were washed three times by PBS before fixing with paraformaldehyde 4% for 10 min. Subsequently, the nuclei of keratinocytes were stained with DAPI (4′,6-diamidino-2-phenylindole; Invitrogen, Karlsruhe, Germany) for 5 min and washed three times with PBS to remove the unspecific DAPI stain. Fluorescent microscopy was performed with Leica DM5500 B Upright microscope. Images were captured with Leica DFC360 FX high-sensitivity monochrome digital camera.

### Electron microscopy

*S*. *aureus* strains USA300 (wild type) and USA300Δ*lpl* were fixed with 2,5% glutaraldehyde and 1% Osmium tetroxide for 45 minutes each, dehydrated and embedded in Epon 812 according to standard procedures [63]. For better visualization of membranes cells were treated with 0,1% Tannin according to the protocol of Gelderblom et al. [64] prior to dehydration. 70 nm ultrathin sections were cut and stained with 2% uranyl acetate and 1% lead citrate. Preparations were examined in a CEM 902 electron microscope (Zeiss) and micrographs taken with TRS camera using Olympus iTEM 5.1 software.

### Transcriptional analysis of *lpl* cluster

USA300 cultured in BM medium was harvested at 3 and 6 h. Total RNA was isolated following our previous study [65]. The qualification and quantity of RNA were determined by agarose gel electrophoresis and photometric measurements via NanoDrop apparatus. Total RNA was subsequently transferred onto a nylon membrane by using a vacuum blotter for 4 h. The RNA was cross-linked to the membrane by UV for 1 min. As control 16S and 23S rRNA bands were visualized by staining with methylene blue. Digoxigenin-labeled RNA probes of three genes in *lpl* cluster (USA300 0410, 0417 and 0420) were used to detect gene-specific hybridization by using ChemiDoc apparatus (Bio-Rad). RNA probes were prepared by using PCR with T7 RNA polymerase. The forward primers and reversed primers, the latter containing a T7 RNA polymerase recognition site at the 5’ end, are listed in S4 Table.

### Statistical analysis

Student's t-tests or analysis of variance (ANOVA) and Mann-Whitney test were employed when appropriate to compare the difference of means. All the statistical analysis was performed by using SPSS v.19 or GraphPad Prism and the significant level was set at *P* value of less than 0.05.

### Ethics statement

Buffy coats were obtained from the transfusion medicine department of the University of Bonn. Informed written consent for the blood donation is obtained at the transfusion unit. Minors are not allowed to donate blood. We received no personal information nor had access to any other type of information on the donors. The buffy coats remained strictly anonymous. The use of peripheral blood leukocytes isolated from buffy coats for this project was approved by the local ethics committee of the medical faculty of the University of Bonn (approval number 36/12).

The use of human skin tissue was approved by the medical ethical committee of the University of Tübingen and was performed in accordance with the Declaration of Helsinki principles. The use of human skin tissue was approved by the medical ethical committee of the University of Tübingen and was performed in accordance with the Declaration of Helsinki principles. The identification number of this approval is 331/2010BO2. All samples were anonymized and written consent was given to the physician in charge (the routine circumcisions were performed at the Loretto Clinic by Dr. med. Frunder).

The mouse skin belongs to the animal studies that were approved by the Regierungspräsidium Tübingen. During the tape-stripping procedure to prime the mouse skin for *S*. *aureus* infection the animals were anesthetized during the procedure. The identification number of this approval is HT1/12. For murine kidney abscess model, all the animal studies were approved by the local government of Franconia, Germany (approval number 55.2–2531.01-06/12) and performed in strict accordance with the guidelines for animal care and experimentation of German Animal Protection Law and the DIRECTIVE 2010/63/EU of the EU.

## Supporting Information

S1 Fig Northern blot analysis of *lpl* cluster. (A) Shows the *lpl* gene cluster and the arrows below indicated the four detectable transcriptional fragments of the *lpl* cluster. (B) Growth curve of USA300 in BM medium; the two time points of harvesting the total RNA at 3 and 6 h are indicated by affow. (C) Northern blots were analyzed with three genes USA300 0410, 0417 and 0420. Both 16S and 23S rRNA were used as a control, but only the 23S rRNA is shown.
(TIF)

S2 Fig Production of TNF-α and IL-6 by Mono Mac 6 cells infected with different MOI of *S*. *aureus* USA300 clones. USA300 wt, its Δ*lpl* mutant and the complemented mutant were cultured in TSB medium for 16 hours and used to infect 10^6^ Mono Mac 6 cells with different multiplicities of infection (MOI) of 15:1, 30:1 and 75:1. (A) TNF-α and (B) IL-6 levels were determined in the supernatant by ELISA after 4 and 24 h of stimulation, respectively. The experiments were conducted 3 times and each time was performed in duplicate. Error bars indicate standard error.
(TIF)

S3 Fig Time-dependence of TNF-α and IL-6 production by Mono Mac 6 cells infected with *S*. *aureus* USA300 clones. USA300 wt, its Δ*lpl* mutant and the complemented mutant were cultured in TSB medium for 16 hours and used to infect 10^6^ Mono Mac 6 cells with multiplicity of infection (MOI) of 30:1. The cytokine levels were measured in the supernatants by ELISA after 4 h, 8 h, 16 h and 24 h of stimulation: (A) TNF-α and (B) IL-6 production.
(TIF)

S4 Fig Survival of Mono Mac 6 cells when infected with *S*. *carnosus* and *S*. *aureus*. The 10^6^ Mono Mac 6 cells were infected with *S*. *aureus* USA300 and *S*. *carnosus* cultured in TSB medium for 16 hours with MOI 30. The unstimulated cells were shown as controls. After 4 and 24 hours of stimulation, the numbers of live Mono Mac 6 cells were counted by using Neubauer Chamber. The experiments were conducted in quadruplicate. The difference among unstimulated cells and *S*. *carnosus*- and *S*. *aureus*-stimulated cells was not significant by using analysis of variance (ANOVA).
(TIF)

S5 Fig Unlipided Lpl1 did not stimulate TNF-α production of Mono Mac 6 cells. (A) SDS-PAGE with purified Lpl1-his (+sp) on the left and purifiedLpl1-his (-sp) on the right. (B) TNF-α production was determined by stimulation of 10^6^ Mono Mac 6 cells with different amounts (200 ng, 500 ng and 1 μg) of purified Lpl1-his (-sp). Unstimulation was considered as negative control and 200 ng of P3C as positive control.
(TIF)

S6 Fig Structure of the lipoprotein cluster in different clonal complexes (CC). The *S*. *aureus* strains in the same clonal complexes share the same structure of lipoprotein cluster and the types of νSaα pathogenic island. Different clonal complexes have different types of νSaα pathogenicity island, except the CC5 and CC8 which contain the most complicated lipoprotein cluster and share the type I of νSaα. The fragments in the black color are flanking genes and the lipoprotein genes are in gray color. The lipoprotein genes with the same pattern show more than 80% of similarity.
(TIF)

S7 Fig Transmission electron microscopic (TEM) images of USA300 and USA300 Δ*lpl*. Comparison of the cell surface between USA300 (right) and Δ*lpl* (left). The samples were taken at 16 h cultured in TSB medium. Scale bar: 200 nm.
(TIF)

S1 Table Strains and plasmids used in this work. (DOCX)

S2 Table Primers used in this study. (DOCX)

S3 Table Primer sequences used for real-time PCR analysis. (DOCX)

S4 Table Primer sequences used for northern blot. (DOCX)
