# Correction: The νSaα Specific Lipoprotein Like Cluster (*lpl*) of *S*. *aureus* USA300 Contributes to Immune Stimulation and Invasion in Human Cells

**DOI:** 10.1371/journal.ppat.1005189

**Published:** 2015-09-25

**Authors:** Minh Thu Nguyen, Beatrice Kraft, Wenqi Yu, Dogan Doruk Demircioglu, Tobias Hertlein, Marc Burian, Mathias Schmaler, Klaus Boller, Isabelle Bekeredjian-Ding, Knut Ohlsen, Birgit Schittek, Friedrich Götz

The fourth author's name is spelled incorrectly. The correct name is: Dogan Doruk Demircioglu. The correct citation is: Nguyen MT, Kraft B, Yu W, Demircioglu DD, Hertlein T, Burian M, et al. (2015) The νSaα Specific Lipoprotein Like Cluster (lpl) of S. aureus USA300 Contributes to Immune Stimulation and Invasion in Human Cells. PLoS Pathog 11(6): e1004984. doi:10.1371/journal.ppat.1004984
