# Supplementary figures and images for: The νSaα Specific Lipoprotein Like Cluster (lpl) of S. aureus USA300 Contributes to Immune Stimulation and Invasion in Human Cells
Source: PLoS Pathog. 2015 Jun 17;11(6):e1004984. doi: 10.1371/journal.ppat.1004984 (PMC4470592; doi:10.1371/journal.ppat.1004984)

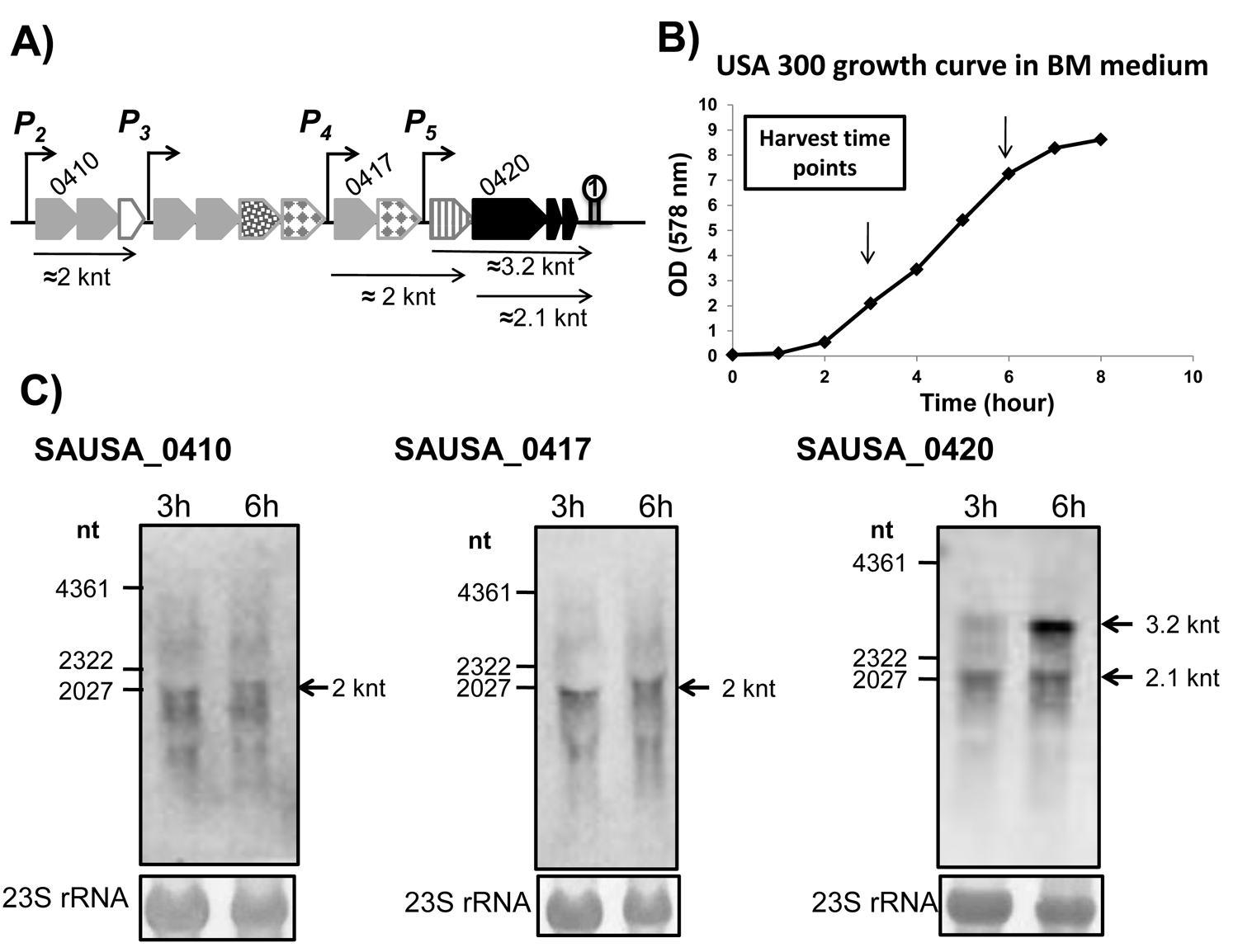

Supplement: S1 Fig — (A) Shows the lpl gene cluster and the arrows below indicated the four detectable transcriptional fragments of the lpl cluster. (B) Growth curve of USA300 in BM medium; the two time points of harvesting the total RNA at 3 and 6 h are indicated by affow. (C) Northern blots were analyzed with three genes USA300 0410, 0417 and 0420. Both 16S and 23S rRNA were used as a control, but only the 23S rRNA is shown. (TIF) [file ppat.1004984.s001.tif]

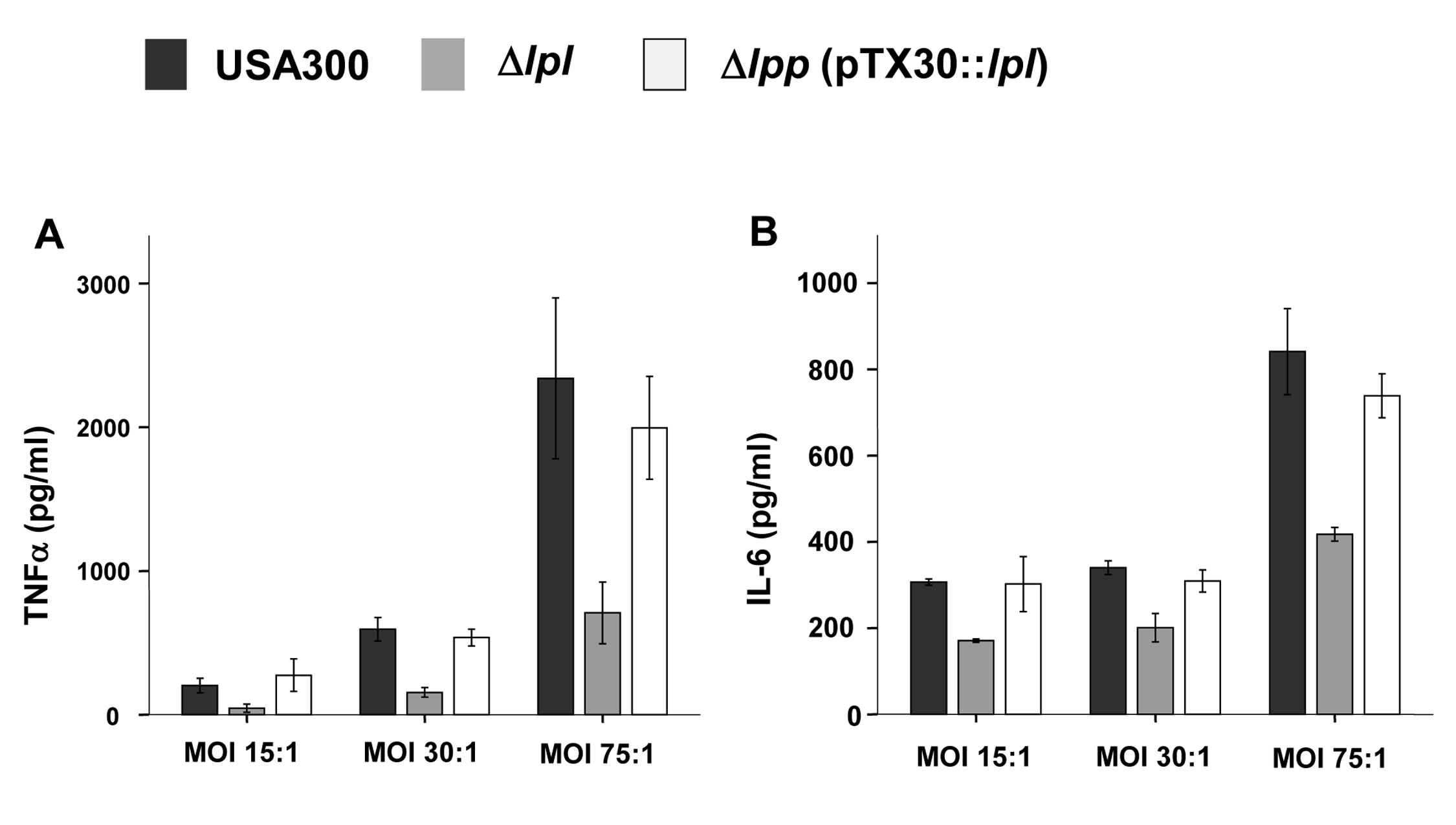

Supplement: S2 Fig — USA300 wt, its Δlpl mutant and the complemented mutant were cultured in TSB medium for 16 hours and used to infect 106 Mono Mac 6 cells with different multiplicities of infection (MOI) of 15:1, 30:1 and 75:1. (A) TNF-α and (B) IL-6 levels were determined in the supernatant by ELISA after 4 and 24 h of stimulation, respectively. The experiments were conducted 3 times and each time was performed in duplicate. Error bars indicate standard error. (TIF) [file ppat.1004984.s002.tif]

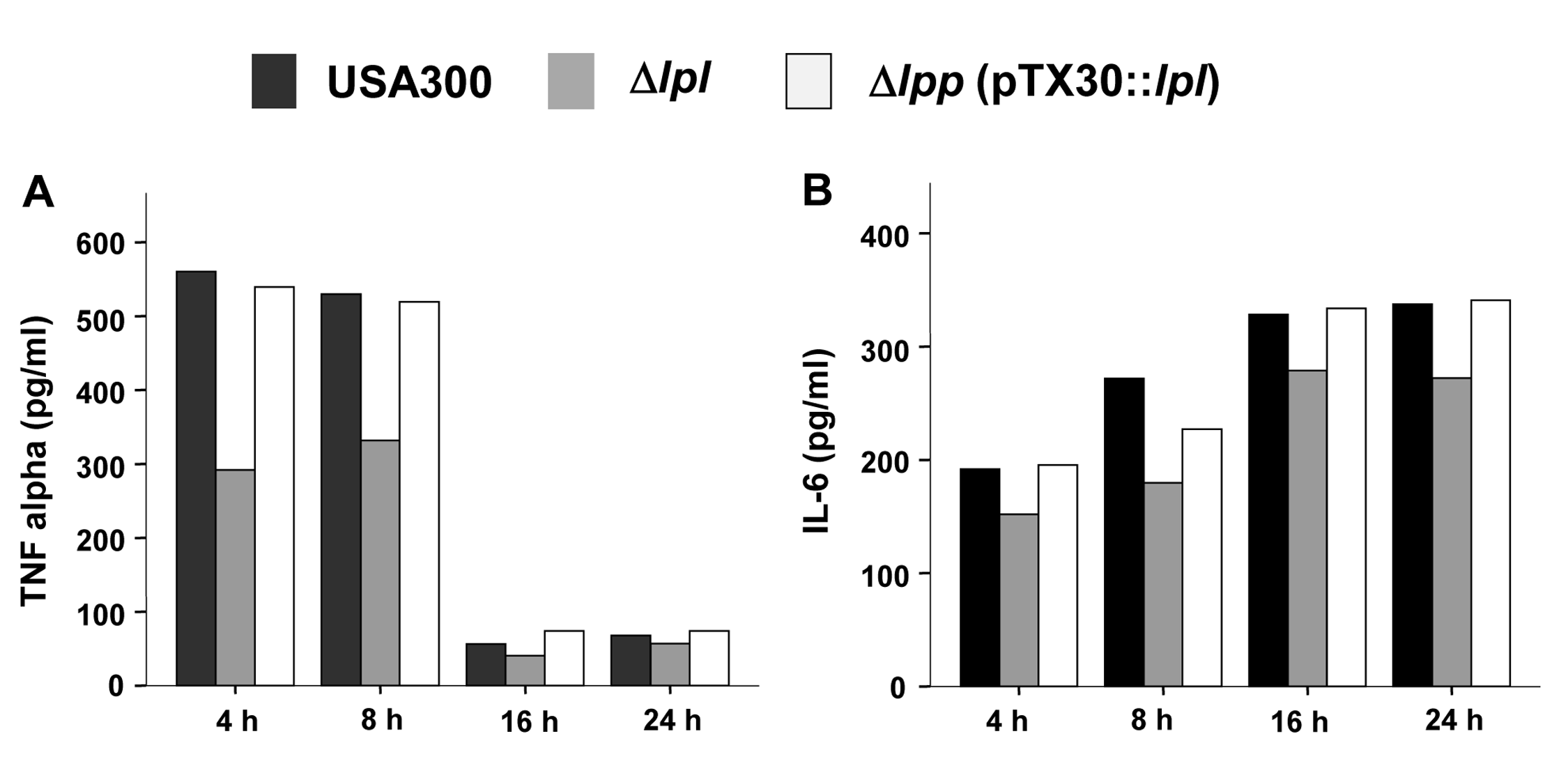

Supplement: S3 Fig — USA300 wt, its Δlpl mutant and the complemented mutant were cultured in TSB medium for 16 hours and used to infect 106 Mono Mac 6 cells with multiplicity of infection (MOI) of 30:1. The cytokine levels were measured in the supernatants by ELISA after 4 h, 8 h, 16 h and 24 h of stimulation: (A) TNF-α and (B) IL-6 production. (TIF) [file ppat.1004984.s003.tif]

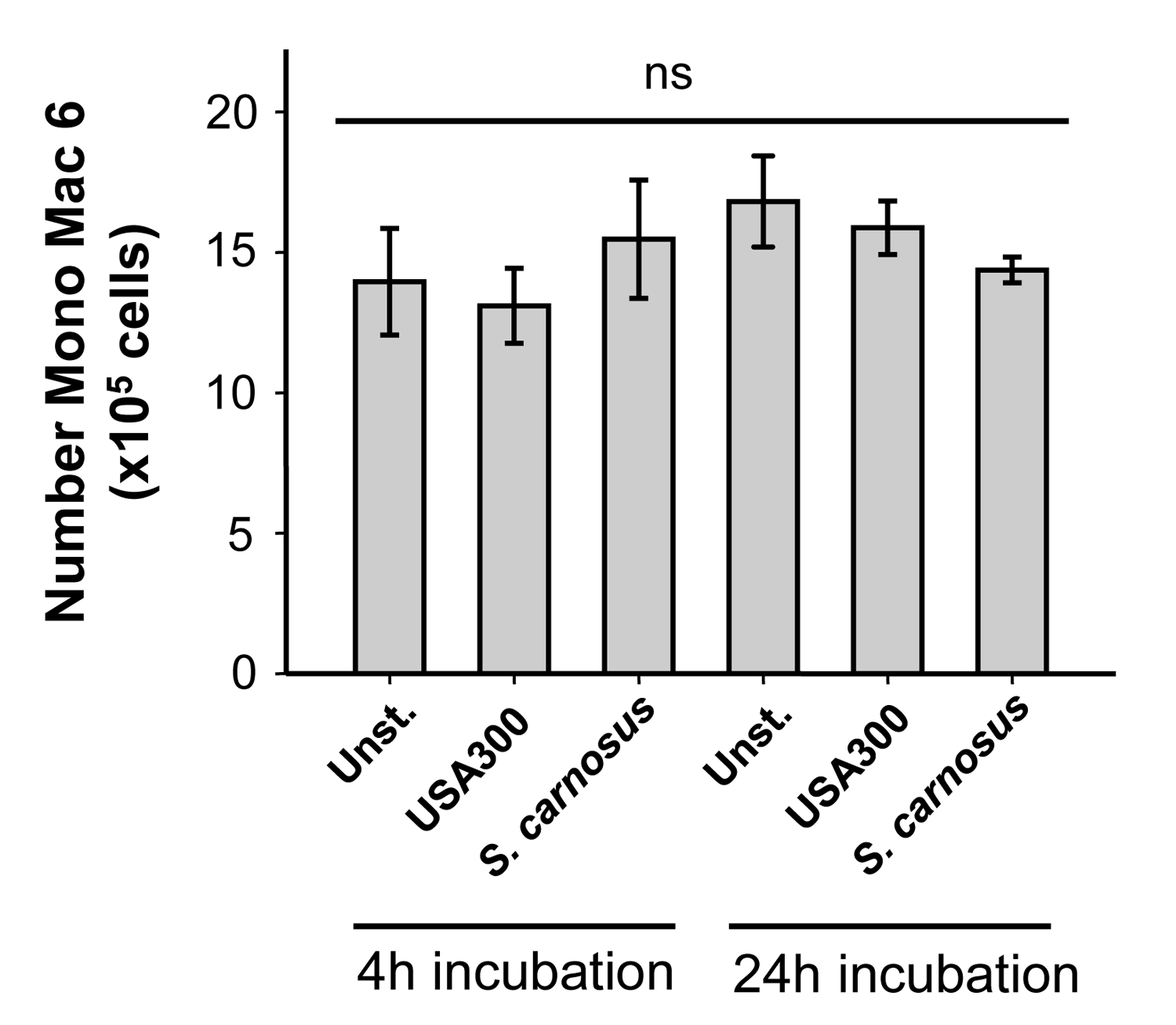

Supplement: S4 Fig — The 106 Mono Mac 6 cells were infected with S. aureus USA300 and S. carnosus cultured in TSB medium for 16 hours with MOI 30. The unstimulated cells were shown as controls. After 4 and 24 hours of stimulation, the numbers of live Mono Mac 6 cells were counted by using Neubauer Chamber. The experiments were conducted in quadruplicate. The difference among unstimulated cells and S. carnosus- and S. aureus-stimulated cells was not significant by using analysis of variance (ANOVA). (TIF) [file ppat.1004984.s004.tif]

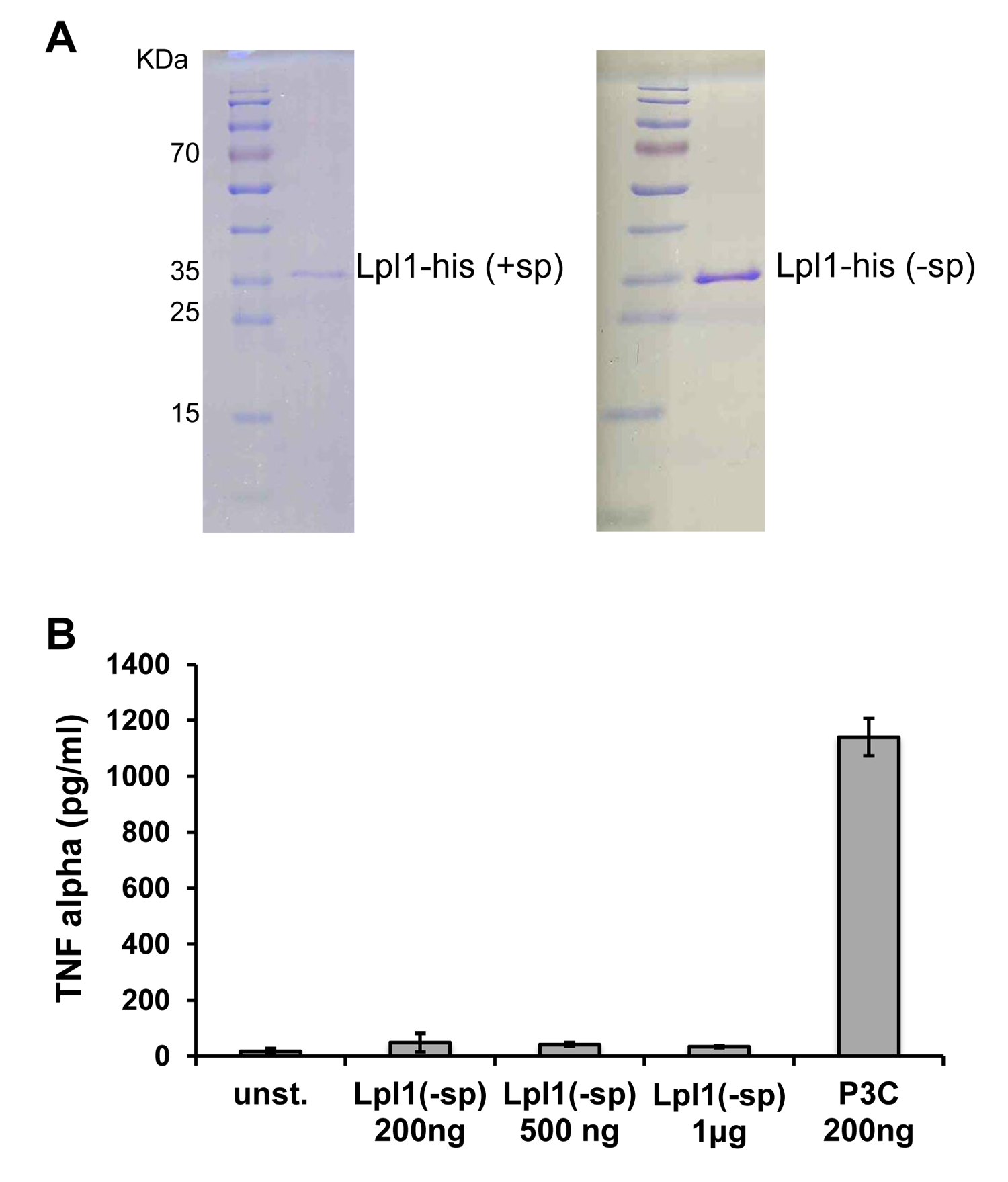

Supplement: S5 Fig — (A) SDS-PAGE with purified Lpl1-his (+sp) on the left and purifiedLpl1-his (-sp) on the right. (B) TNF-α production was determined by stimulation of 106 Mono Mac 6 cells with different amounts (200 ng, 500 ng and 1 μg) of purified Lpl1-his (-sp). Unstimulation was considered as negative control and 200 ng of P3C as positive control. (TIF) [file ppat.1004984.s005.tif]

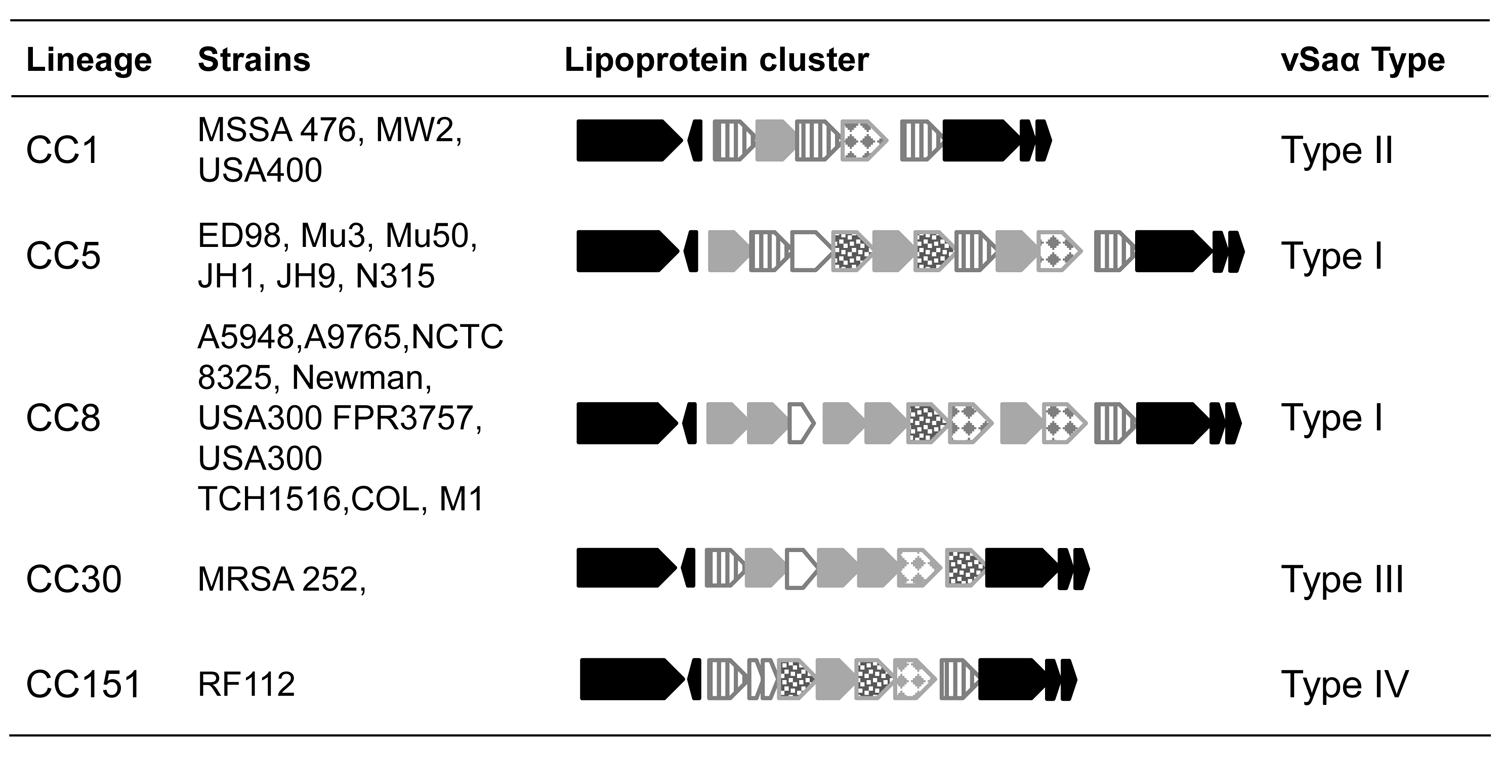

Supplement: S6 Fig — The S. aureus strains in the same clonal complexes share the same structure of lipoprotein cluster and the types of νSaα pathogenic island. Different clonal complexes have different types of νSaα pathogenicity island, except the CC5 and CC8 which contain the most complicated lipoprotein cluster and share the type I of νSaα. The fragments in the black color are flanking genes and the lipoprotein genes are in gray color. The lipoprotein genes with the same pattern show more than 80% of similarity. (TIF) [file ppat.1004984.s006.tif]

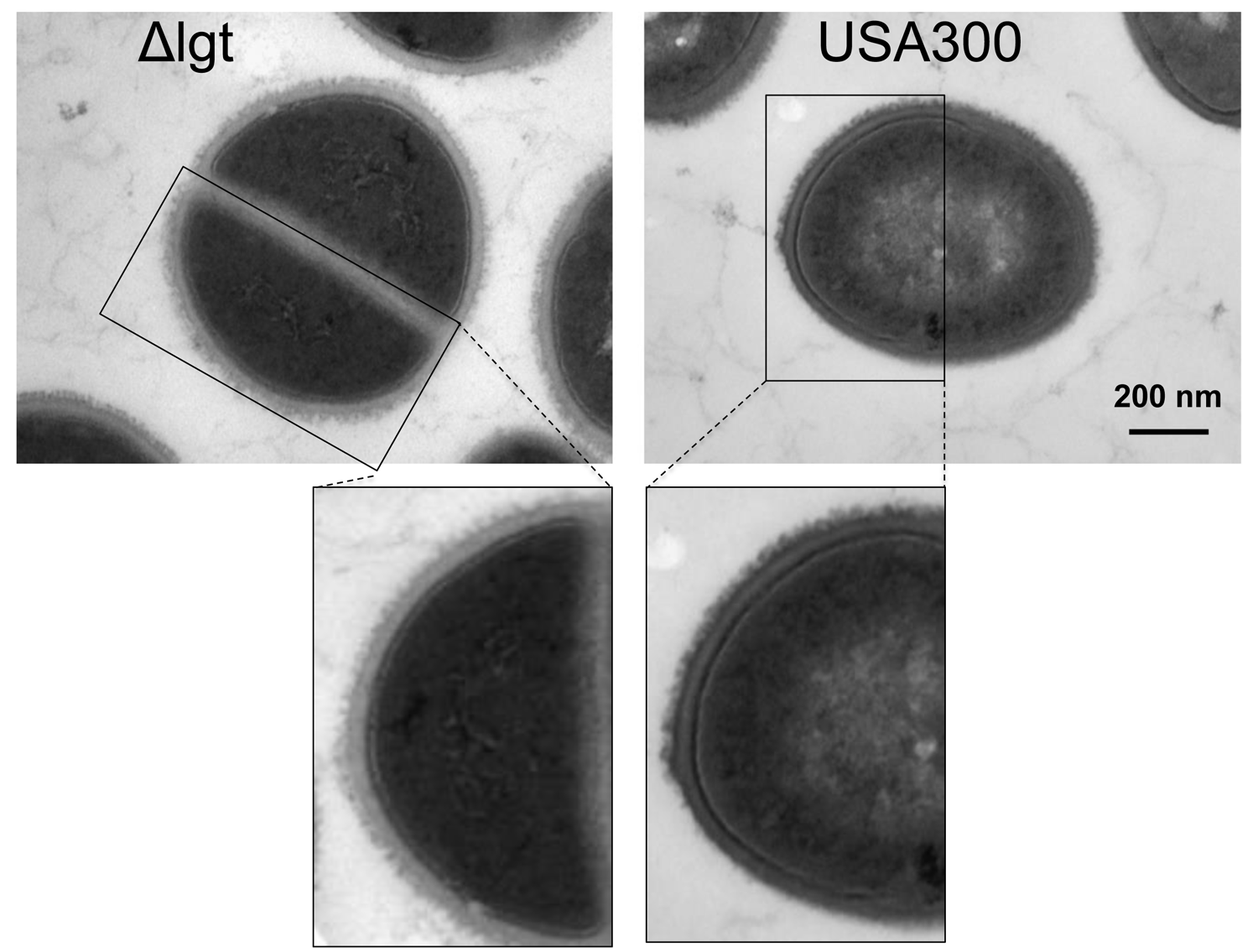

Supplement: S7 Fig — Comparison of the cell surface between USA300 (right) and Δlpl (left). The samples were taken at 16 h cultured in TSB medium. Scale bar: 200 nm. (TIF) [file ppat.1004984.s007.tif]
