# Supplementary material for: The νSaα Specific Lipoprotein Like Cluster (lpl) of S. aureus USA300 Contributes to Immune Stimulation and Invasion in Human Cells
Source: PLoS Pathog. 2015 Jun 17;11(6):e1004984. doi: 10.1371/journal.ppat.1004984 (PMC4470592; doi:10.1371/journal.ppat.1004984)
Supplement: S1 Table — (DOCX) [file ppat.1004984.s008.docx]

**S1 Table. Strains and plasmids used in this work.**

| **Strains or Plasmids** | **Description** | **Reference** |
| --- | --- | --- |
| **Strains** |  |  |
| *E.coli* |  |  |
| DH5-α | Laboratory strain | [1] |
| *S. aureus* |  |  |
| USA300 (FPR3757) | Laboratory strain |  |
| USA300 Δ*lpl* | USA300*Δlpl* markerless deletion | This study |
| SA113 | Mutant of strain RN1 (NTCC8325), r- m- | [2] |
| SA113 Δ*lgt* | SA113*Δlgt::ermB* | [2] |
| HG003 | Mutant of strain RN1 (NTCC8325), *rsbU&tcaR* repaired | [3] |
| RN4220 | NCTC8325-4 derivative, acceptor of foreign DNA | [4] |
| *S.carnosus* |  |  |
| MT300 | Laboratory strain |  |
| **Plasmids** |  |  |
| pTX30 | Xylose inducible expression vector | [5] |
| pTX-*lpl* | Complementary plasmid | This study |
| pTX30::*lpl1*-his | Overexpression of the first tandem *lpl* | This study |
| pCX15 | Xylose inducible expression vector | [6] |
| pCX15::0420÷0422 |  | This study |
| pBT2 | Knock-out plamid | [7] |
| pBT2Δ*lpl* | Knock-out plasmid for replacement of *lpl* | This study |
| pRAB1 | cat bla, P_pagA_-cre; pBT2 derivative; expression of cre in staphylococci | [8] |

Reference List

1. Schlag M, Biswas R, Krismer B, Kohler T, Zoll S, et al. (2010) Role of staphylococcal wall teichoic acid in targeting the major autolysin Atl. MolMicrobiol 75: 864-873.

2. Stoll H, Dengjel J, Nerz C, Götz F (2005) *Staphylococcus aureus* deficient in lipidation of prelipoproteins is attenuated in growth and immune activation. Infect Immun 73: 2411-2423.

3. Herbert S, Ziebandt AK, Ohlsen K, Schäfer T, Hecker M, et al. (2010) Repair of global regulators in *Staphylococcus aureus* 8325 and comparative analysis with other clinical isolates. Infect Immun.

4. Iordanescu S, Surdeanu M (1976) Two restriction and modification systems in *Staphylococcus aureus* NCTC8325. JGenMicrobiol 96: 277-281.

5. Müller P, Müller-Anstett M, Wagener J, Gao Q, Kaesler S, et al. (2010) The *Staphylococcus aureus* lipoprotein SitC colocalizes with Toll-like receptor 2 (TLR2) in murine keratinocytes and elicits intracellular TLR2 accumulation. InfectImmun 78: 4243-4250.

6. Strauss A, Götz F (1996) *In vivo* immobilization of enzymatically active polypeptides on the cell surface of *Staphylococcus carnosus*. Mol Microbiol 21: 491-500.

7. Brückner R (1992) A series of shuttle vectors for *Bacillus subtilis* and *Escherichia coli*. Gene 122: 187-192.

8. Leibig M, Krismer B, Kolb M, Friede A, Götz F, et al. (2008) Marker removal in staphylococci via Cre recombinase and different lox sites. ApplEnvironMicrobiol 74: 1316-1323.
