# Supplementary material for: The νSaα Specific Lipoprotein Like Cluster (lpl) of S. aureus USA300 Contributes to Immune Stimulation and Invasion in Human Cells
Source: PLoS Pathog. 2015 Jun 17;11(6):e1004984. doi: 10.1371/journal.ppat.1004984 (PMC4470592; doi:10.1371/journal.ppat.1004984)
Supplement: S2 Table — (DOCX) [file ppat.1004984.s009.docx]

**S2 Table. Primers used in this study.**

| **Primer name** | **Sequence 5’-3’** |
| --- | --- |
| Fr_up (EcoRI) | CCGGAATTCAGCTTTGATGTAGATCATG |
| Re_up (Xhol) | GCGCTCGAGTGTATATTTCTCTTTTACGTTACCA |
| Fr_down (XbaI) | CCGTCTAGACTACATATCATACTACTAAATAAAGCG |
| Re_down (BamHI) | GCGGGATCCTAAACGGCAAATCGCTGTAA GATCA |
| Fr_erm(XbaI) | CGCTCTAGA*TACCGTTCGTATAATGTATGCTATACGAAGTTAT*AGTATTGTCCGAGAGTGATTGGTCT |
| Re_erm(XhoI) | CGCCTCGAG*TACCGTTCGTATAGCATACATTATACGAAGTTAT*CTCCTTGGAAGCTGTCAGTAGTATACCT |
| Fr_0410 (BamHI) | GCGGGATCCTGAAAAGAGAATATAAATGAAGTAT |
| Re_0414(Sal1) | GCCGTCGACACACCCTAATATTTAATTATCAGTAC |
| Fr_0415 (Sal1) | GCCGTCGACATGGAATATCTAAAAAGGCTTGCAT |
| Re_0418 (AvrII) | CGGCCTAGGTTATCAAACAAAACTAACTTATTCA |
| Fr_0419 (AvrII) | CGCCCTAGGAGTTGAATAATCACTATTTTAATAAG |
| Re_ 0422 (SacI) | CCGGAGCTCTTAGTAGTATGATATGTAGTTTAGC |
| Fr_0420 (BamHI) | CGCGGATCCAGGAGGTTGAGAATGTTGAGTAGGAAGTATAA |
| Re_0422 (XbaI) | CCGTCTAGATTAGTAGTATGATATGTAGTTTAGC |
| Re_0424 | CTTCATTCAACTTATCGTGCTGGTC |
| Fr_lpl1(+sp) | GCGGGATCCTGAA**AGGAGG**ATATAAATGAAGTAT |
| Fr_lpl1(-sp) | GCGGGATCCTGAA**AGGAGG**ATATAAATGTGTGGCAAAGGTAATGAAACAAAAG |
| Re_lpl1-his (SacI) | CCGGAGCTCCTA*ATGATGATGATGATGATG*TTTTTTCGCTGGTTTATAACTTAAA |
