# Supplementary material for: The νSaα Specific Lipoprotein Like Cluster (lpl) of S. aureus USA300 Contributes to Immune Stimulation and Invasion in Human Cells
Source: PLoS Pathog. 2015 Jun 17;11(6):e1004984. doi: 10.1371/journal.ppat.1004984 (PMC4470592; doi:10.1371/journal.ppat.1004984)
Supplement: S3 Table — (DOCX) [file ppat.1004984.s010.docx]

**S3 Table. Primer sequences used for real-time PCR analysis.**

| **Species** | **Genes** | **Sequence** |
| --- | --- | --- |
| Human | β-actin (forward) | 5’-ttgttacaggaagtcccttgcc-3’ |
|  | β -actin (reverse) | 5’-atgctatcacctcccctgtgtg-3’ |
|  | TNF-α (forward) | 5’-cccaggcagtatcttc-3’ |
|  | TNF-α (reverse) | 5’-agctgcccctgcttga-3’ |
|  | IL-1β (forward) | 5’-ctgtcctgcggaaaga-3’ |
|  | IL-1β (reverse) | 5’-ttgggtaatttctaca-3’ |
|  | RNase7 (forward) | 5’-gaagaccaagcgcaaagc-3’ |
|  | RNase7 (reverse) | 5’-cagcagaagcagcagaagg-3’ |
