# Supplementary material for: The νSaα Specific Lipoprotein Like Cluster (lpl) of S. aureus USA300 Contributes to Immune Stimulation and Invasion in Human Cells
Source: PLoS Pathog. 2015 Jun 17;11(6):e1004984. doi: 10.1371/journal.ppat.1004984 (PMC4470592; doi:10.1371/journal.ppat.1004984)
Supplement: S4 Table — (DOCX) [file ppat.1004984.s011.docx]

**S4 Table. Primer sequences used for northern blot.**

| **Genes** | **Sequence** |
| --- | --- |
| SAUSA_0410 (forward) | 5’-cagagagacgtgaagcgatg-3’ |
| SAUSA_0410 (reverse) | 5’-ctaatacgactcactatagggagacagaactgcctttcaggtcc-3’ |
| SAUSA_0417 (forward) | 5’-cgaggagttatacgacaaag-3’ |
| SAUSA_0417 (reverse) | 5’-ctaatacgactcactatagggagatcatcttcatctaccttcgc-3’ |
| SAUSA_0420 (forward) | 5’-gaaagcgtcatacgaagttg-3’ |
| SAUSA_0420 (reverse) | 5’-ctaatacgactcactatagggagactcctccatttgtagtcatc -3’ |
